# Tube-dwelling in early animals exemplified by Cambrian scalidophoran worms

**DOI:** 10.1186/s12915-021-01172-4

**Published:** 2021-11-12

**Authors:** Deng Wang, Jean Vannier, Cédric Aria, Jie Sun, Jian Han

**Affiliations:** 1 Shaanxi Key Laboratory of Early Life and Environments, State Key Laboratory of Continental Dynamics, Department of Geology, Northwest University, Xi’an, 710069 China; 2 Univ Lyon, Univ Lyon 1, ENSL, CNRS, LGL-TPE, F-69622 Villeurbanne, France

**Keywords:** Priapulida, Cambrian tubicolous worms, *Selkirkia*, Palaeobiology, Chengjiang Lagerstätte

## Abstract

**Background:**

The radiation of ecdysozoans (moulting animals) during the Cambrian gave rise to panarthropods and various groups of worms including scalidophorans, which played an important role in the elaboration of early marine ecosystems. Although most scalidophorans were infaunal burrowers travelling through soft sediment at the bottom of the sea, *Selkirkia* lived inside a tube.

**Results:**

We explore the palaeobiology of these tubicolous worms, and more generally the origin and evolutionary significance of tube-dwelling in early animals, based on exceptionally preserved fossils from the early Cambrian Chengjiang Lagerstätte (Stage 3, China) including a new species, *Selkirkia transita* sp. nov. We find that the best phylogenetic model resolves *Selkirkia* as a stem-group priapulid. *Selkirkia* secreted a protective cuticular thickening, the tube, inside which it was able to move during at least part of its life. Partly based on measured growth patterns, we construe that this tube was separated from the trunk during a moulting process that has no direct equivalent in other scalidophorans. Although the ontogeny of *Selkirkia* is currently unknown, we hypothesize that its conical tube might have had the same ecological function and possibly even deep development origin as the lorica, a protective cuticular thickening found in larval priapulids and adult loriciferans. *Selkirkia* is seen as a semi-sedentary animal capable of very shallow incursions below the water/sediment interface, possibly for feeding or during the tube-secreting phase. Brachiopod epibionts previously reported from the Xiaoshiba Lagerstätte (ca. 514 Ma) also presumably occur in *Selkirkia sinica* from Chengjiang (ca. 518 Ma).

**Conclusions:**

Our critical and model-based approach provides a new phylogenetic framework for Scalidophora, upon which to improve in order to study the evolution of morphological characters in this group. Tube-dwelling is likely to have offered *Selkirkia* better protection and anchoring to sediment and has developed simultaneously in other Cambrian animals such as hemichordates, annelids or panarthropods. Often lost in modern representatives in favour of active infaunal lifestyles, tube-dwelling can be regarded as an early evolutionary response of various metazoans to increasing environmental and biological pressure in Cambrian marine ecosystems.

**Supplementary Information:**

The online version contains supplementary material available at 10.1186/s12915-021-01172-4.

## Background

Scalidophora (Priapulida, Kinorhyncha and Loricifera) [1] constitutes a minor part of Ecdysozoa (moulting protostomes), which encompasses more than millions of arthropod species [2]. The diversity of present-day scalidophorans is relatively low with less than 300 described species [2, 3], yet Priapulida, Kinorhyncha and Loricifera all have representatives in the Cambrian [4–7]. Stem-group priapulids represent in fact the most abundant and diverse group of endobenthic worms of Cambrian marine ecosystems [8]. The evolutionary interest of scalidophorans therefore lies (1) in their rich Cambrian fossil record which documents steps in the early assembly of the ecdysozoan body plan and (2) in the crucial role played by this animal group in early marine ecosystems (e.g. bioturbation,[9]). Scalidophorans share a number of basic anatomical features such as an eversible introvert, a tubular annulated trunk, a relatively spacious primary body cavity and a well-developed network of longitudinal, circular and retractor muscles [3]. Scalidophorans with diverse morphologies have been reported from the lowermost Cambrian Kuanchuanpu Formation (ca. 535 Ma), such as *Eokinorhynchus rarus* [10], *Eopriapulites sphinx* (phosphatized microfossils) [11], but the diversity of the group reaches its peak during the early-to-mid-Cambrian. They are particularly diverse and abundant in Burgess Shale-type Lagerstätten such as those of Chengjiang [8], Sirius Passet [12], and the Burgess Shale [4].

Whereas most Cambrian scalidophorans were elongated and flexible worms, selkirkiids lived within a conical tube open at both ends [4]. These enigmatic tubicolous worms were first described from the Burgess Shale Lagerstätte (*Selkirkia columbia* [4, 13]) but occur in other North American localities [14–16] and have been found in several Chinese Lagerstätten such as those of Chengjiang (*Paraselkirkia jinningensis* Hou et al., 1999 [17] = *Selkirkia sinica* Lou et al., 1999 [18]), Xiaoshiba [19] and Kaili [20] (Additional file 1: Text S1 )[4, 14, 16–18, 21–26]. Another alleged selkirkiid genus, *Sullulika*, was reported from the Sirius Passet Lagerstätte in Greenland [27], but its affinity is difficult to ascertain due to the lack of information on its soft anatomy. The nature of the selkirkiid tube has been discussed by several authors [4, 28, 29] but major uncertainties remain concerning its structure, composition, relation to the animal’s body, and whether this tube may be homologous or not with the lorica of loriciferans and larval priapulids. Equally unresolved is the capacity of selkirkiids to possibly move within their tube and through their environment. More generally, through this reinvestigation of *Selkirkia*, we address the significance of tube-dwelling habits in early Cambrian ecosystems.

## Results

### Preservation

**Fig. 1:**
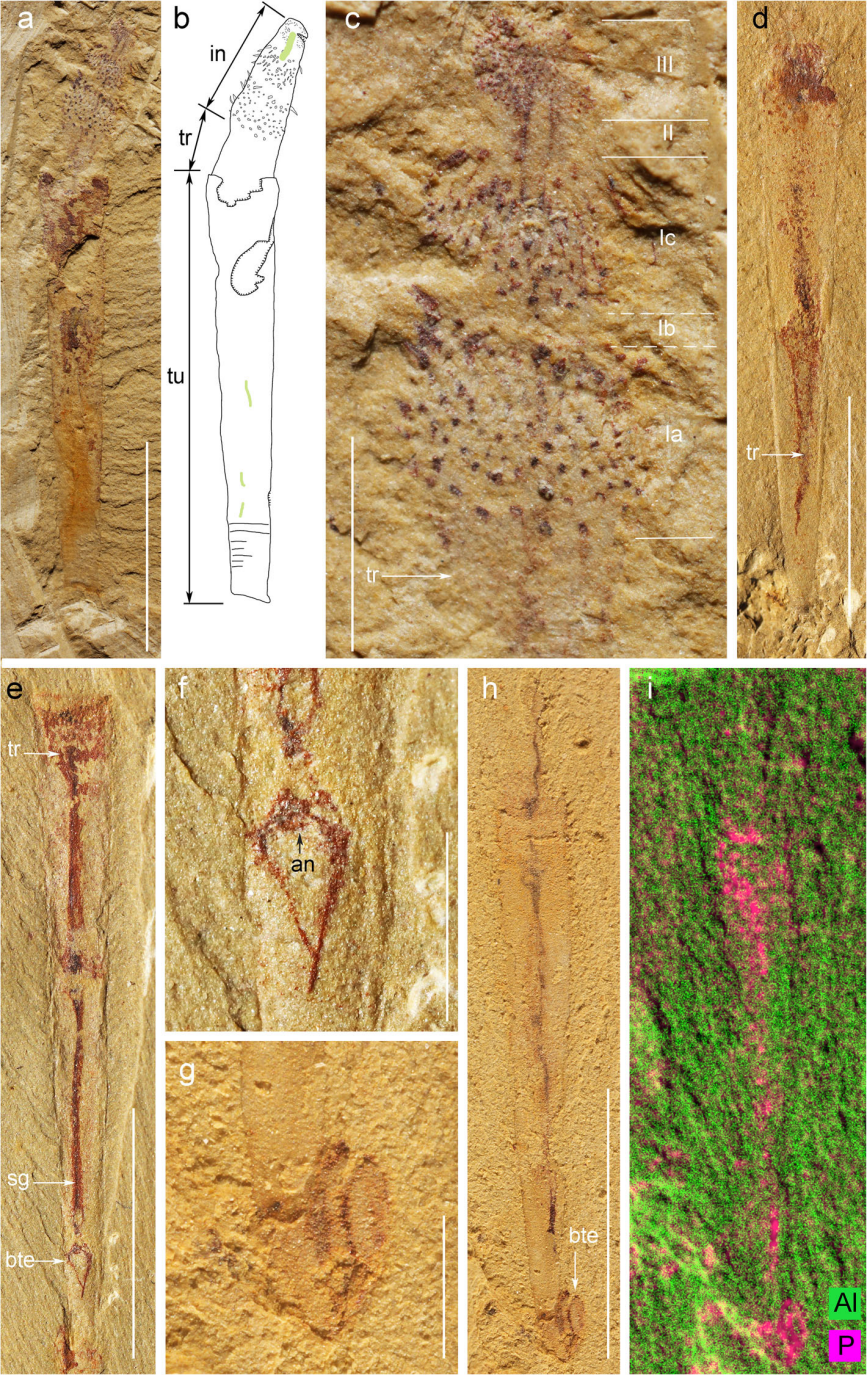
*Selkirkia sinica* from the early Cambrian Chengjiang Lagerstätte: general body plan, trunk and possible paired caudal appendages. **a**–**c** ELI-0002001, *Selkirkia sinica*, general view, line drawing and close-up of introvert. **d** ELI-0002002, showing shrinked remains of the trunk and a tapering trunk end. **e**, **f** ELI-0002003, showing strongly shrinked trunk and bifurcating trunk ends and close up of corresponding structures. **g**–**i** ELI-0002004, showing possible bifurcating structures at the posterior end of the trunk, general view, close-up and X-ray Fluorescence (XRF) image (note that bifurcating structures and trunk both have comparable elemental distribution). Abbreviations: an, anus; bte, possible bifurcated trunk ends; sg, straight gut; tr, trunk. Scale bars represent **a**, **d**, **e** and **h** 5 mm; **c**, **f** and **g** 1 mm

**Fig. 2:**
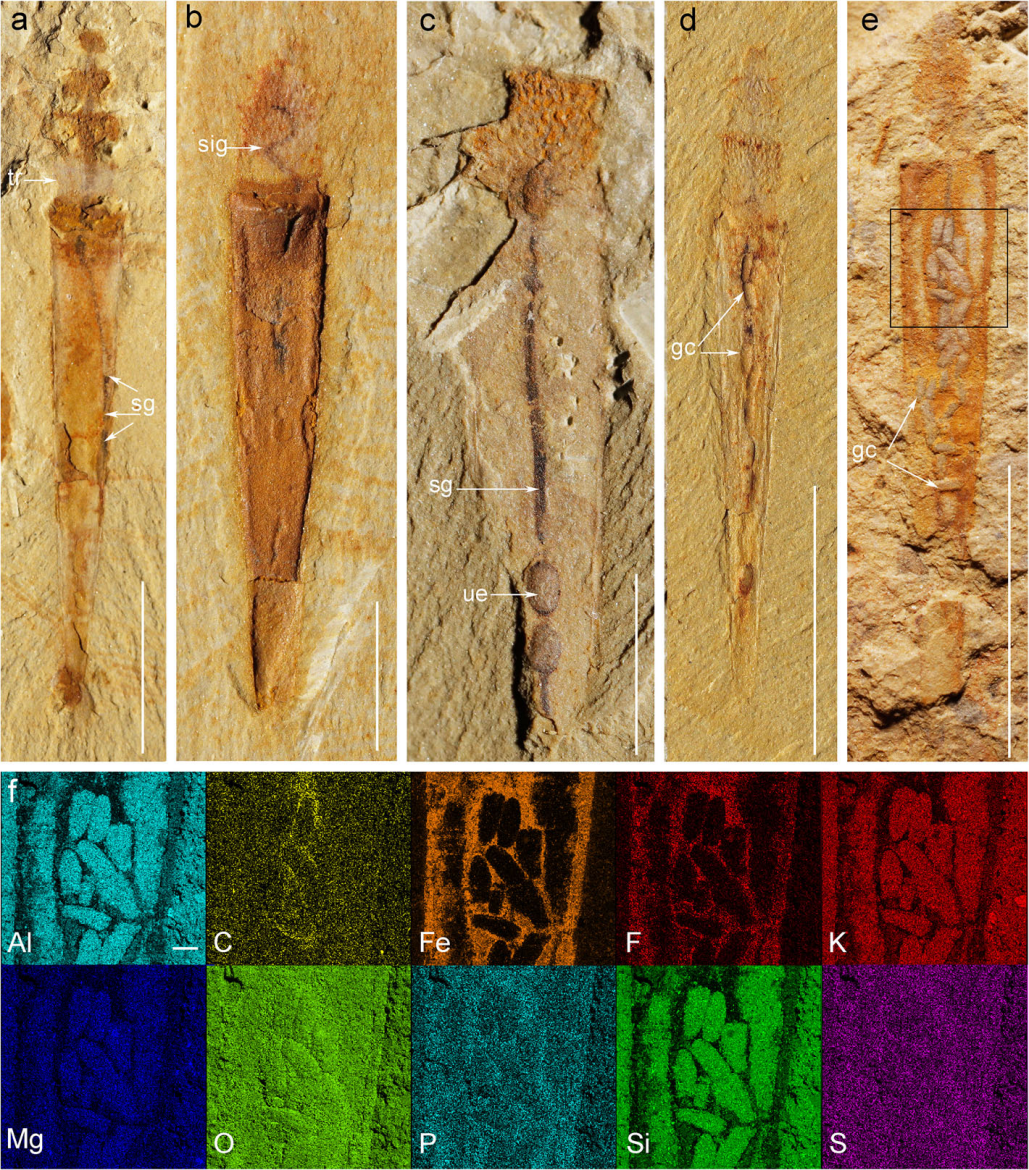
*Selkirkia sinica* from the early Cambrian Chengjiang Lagerstätte: gut tract and gut contents. **a** ELI-00001405, showing fully everted introvert and basal part of trunk protruding beyond the tube opening. **b** ELI-00002012, showing sinuous gut inside introvert. **c** ELI-00002013, showing thin and straight gut tract and, posteriorly, two ovoid elements interpreted as possible unusually large pellets or undigested shells. **d** ELI-00002014, showing fully everted introvert and 3D-preserved aligned pellet-like elements in gut. **e**, **f** ELI-00002015, showing more randomly distributed pellet-like elements in the gut (compared with **d**), elemental maps (see location in **e**). Abbreviations: gc, gut contents; sg, straight gut; sig, sinuous gut; tr, trunk; ue, undigested element (possibly shell). Scale bars represent **a**, **d** and **e** 5 mm; **b**, **c** 2 mm; **f** 200 μm

**Fig. 3:**
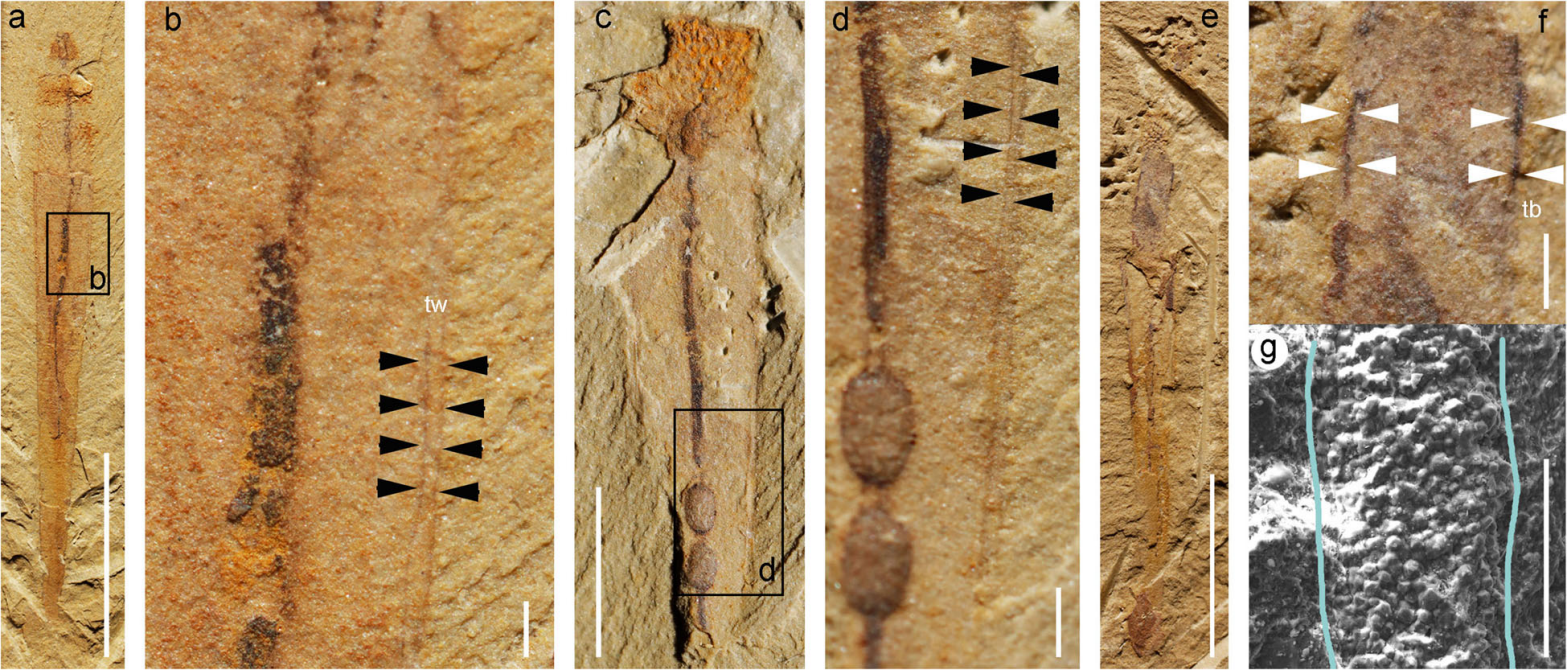
*Selkirkia sinica* from the early Cambrian Chengjiang Lagerstätte: relation between trunk and tube. **a**, **b** ELI-0002016, showing tube wall (external and internal boundaries outlined by very pale coloured layers; see black arrows), general view and close-up. **c**, **d** ELI-0002013, showing tube wall (as in **b**), general view and close-up. **e**–**g** ELI-0002017, showing anterior part of trunk protruding outside the tube; trunk cuticle appears as a thin dark layer (white arrows), SEM image showing tube wall preserved in iron oxides. Abbreviations: tb, trunk boundary; tw, tube wall. Scale bars represent **a**, **e** 5 mm; **c** 2 mm; **d** 1 mm; **f** 400 μm; **b** 200 μm; and **g** 100 μm

**Fig. 4:**
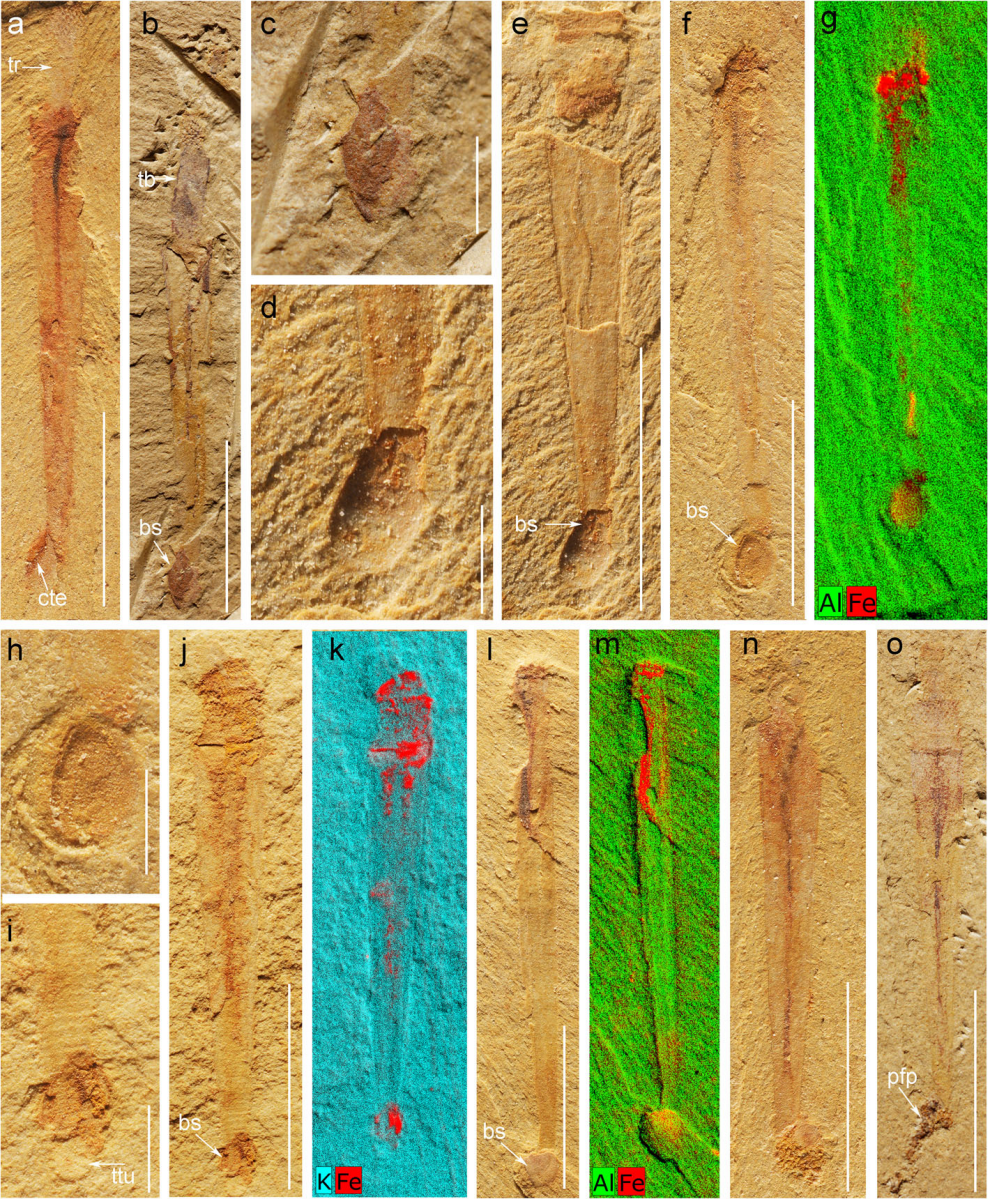
*Selkirkia sinica* from the early Cambrian Chengjiang Lagerstätte: possible brachiopod epibionts and possible faecal pellets. **a** ELI-0002018, showing damaged posterior part of tube. **b**, **c** ELI-0002017, showing protruding part of trunk and possible brachiopod shell near tube end. **d**, **e** ELI-0002019, showing possible brachiopod attached near the posterior end of tube. **f–h** ELI-0002020, showing possible brachiopod attached near the posterior end of tube. **i–k** ELI-0002021, showing possible brachiopod attached to the tube and tube annulations. **l**, **m** ELI-0002022, showing possible brachiopod fragment near the posterior end of tube. **n** ELI-0002023 and **o** ELI-0002024, showing possible faecal pellets released from the posterior opening of the tube (e.g. undigested coarse sediment particles). **g**, **k** and **m** are XRF images to show elemental differences between tube and possible brachiopod. Abbreviations: an, annulations, bs, possible brachiopod shell; cte, cracked tube end; pfp, possible faecal pellets; tb, trunk boundary; tr, trunk; ttu, tapered tube end. Scale bars represent **a**, **b**, **e**, **f**, **j**, **l**, **n** and **o** 5 mm; **c**, **d**, **h** and **i** 1 mm

**Fig. 5:**
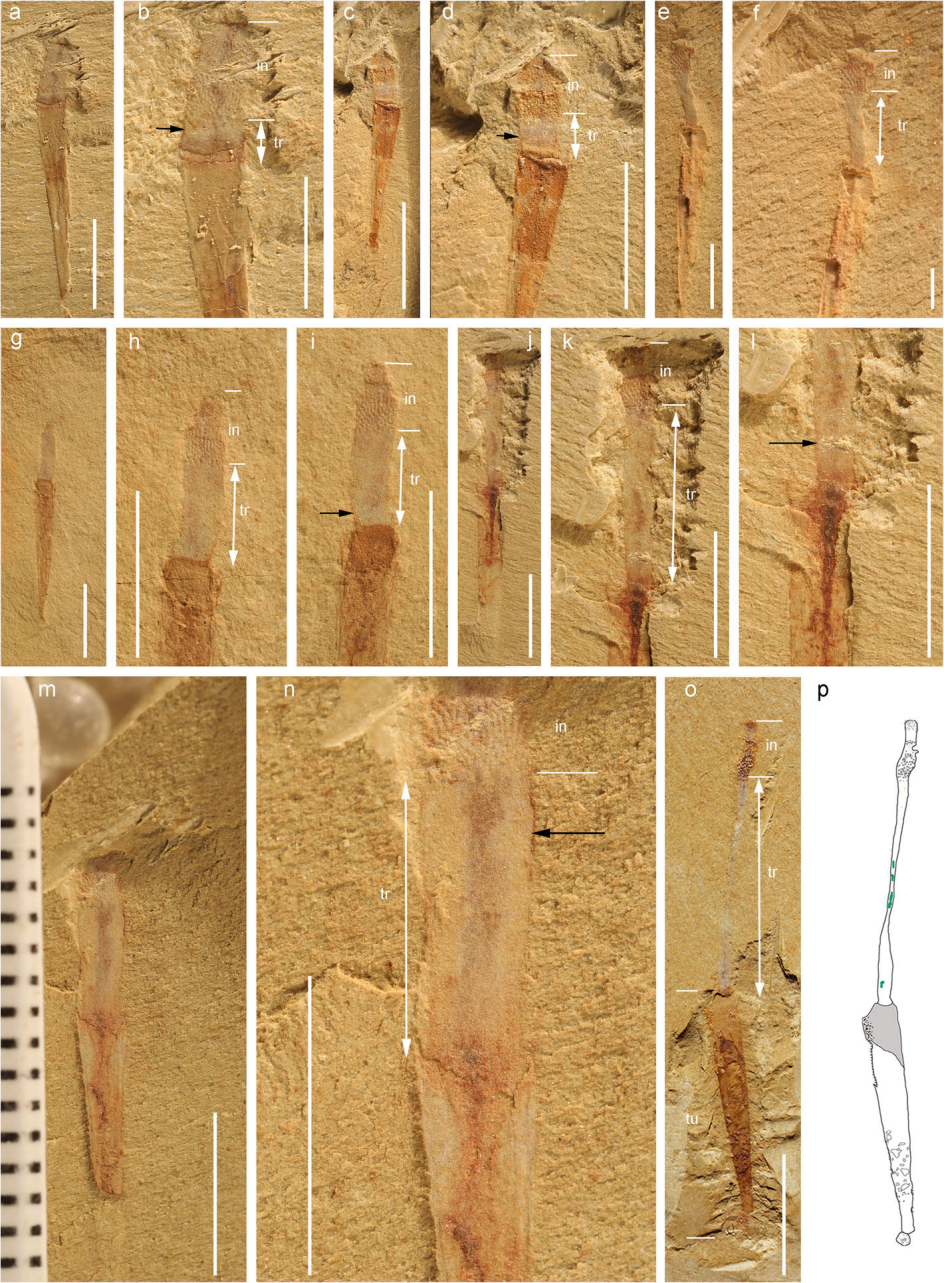
*Selkirkia sinica* from the early Cambrian Chengjiang Lagerstätte: protruding trunk in various length. **a**, **b** ELI-0002005, general view, and close-up (trunk protruding about 1 mm outside tube opening). **c**, **d** ELI-0002006, general view, and close-up (trunk protruding about 2 mm outside tube opening). **e**, **f** ELI-0002007, protruding trunk and distinct gap between trunk and tube (black line), and mineral grains (white arrow). **g–i** ELI-0002008, general view, and close-up (trunk protruding about 3 mm outside tube opening). **h** and **i** represent part and counterpart, respectively. **j–l** ELI-0002009, general view, and close-up (trunk protruding about 7 mm outside tube opening). **m–n** ELI-0002010, general view, and close-up (trunk protruding about 3 mm outside tube opening). **o–p** ELI-0002011, general view, and line drawing (trunk protruding about 10 mm beyond tube opening, almost equivalent to tube length). Black arrows indicate the trunk cuticle, white double arrows indicate the protruding length of the trunk. I, II and III represent Zone I, Zone II and Zone III, respectively. Ia, Ib and Ic represent the subdivisions of Zone I. Green lines and grey area in **p** indicate the gut and inner surface of the tube, respectively. Scale bars represent **a**, **c**, **g**, **j**, **m** and **o** 5 mm; **e** 2 mm; **b**, **d**, **f**, **h**, **i**, **k**, **l** and **n** 1 mm

**Fig. 6:**
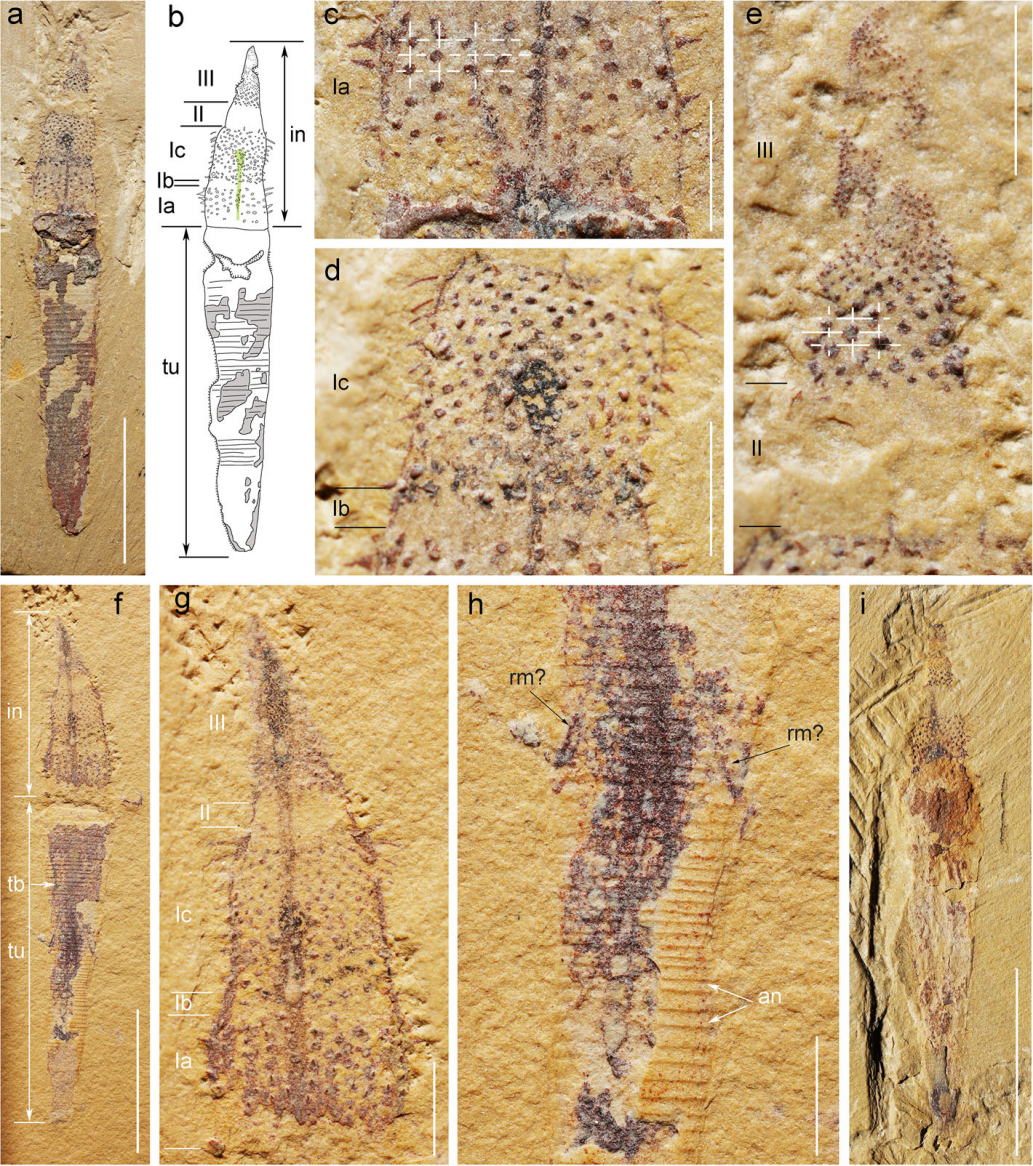
*Selkirkia transita* sp. nov. from the early Cambrian Chengjiang Lagerstätte: general morphology. **a–e** ELI-0000601, holotype, *Selkirkia transita* sp. nov., general view, line drawing, and details of introvert structure and ornament. **f**–**h** ELI-0000602, paratype, showing introvert, possible oblique retractor muscles and annulations along tube. **i** ELI-0000603, paratype, showing part of introvert. Abbreviations: an, annulation; in, introvert; rm?, possible retractor muscles; tb, trunk boundary; tr, trunk; tu, tube. I, II and III represent divisions Zone I, Zone II and Zone III of the introvert, respectively. Ia, Ib, Ic represent the subdivisions of Zone I. Green lines and grey areas in **b** represent the gut and inner surface of the tube, respectively. White dotted lines in **c**, **e** show scalids and pharyngeal teeth arranged in quincunx, respectively. Scale bars represent **a**, **f**, and **i** 5 mm; **c**–**e**, **g** and **h** 1 mm

**Fig. 7:**
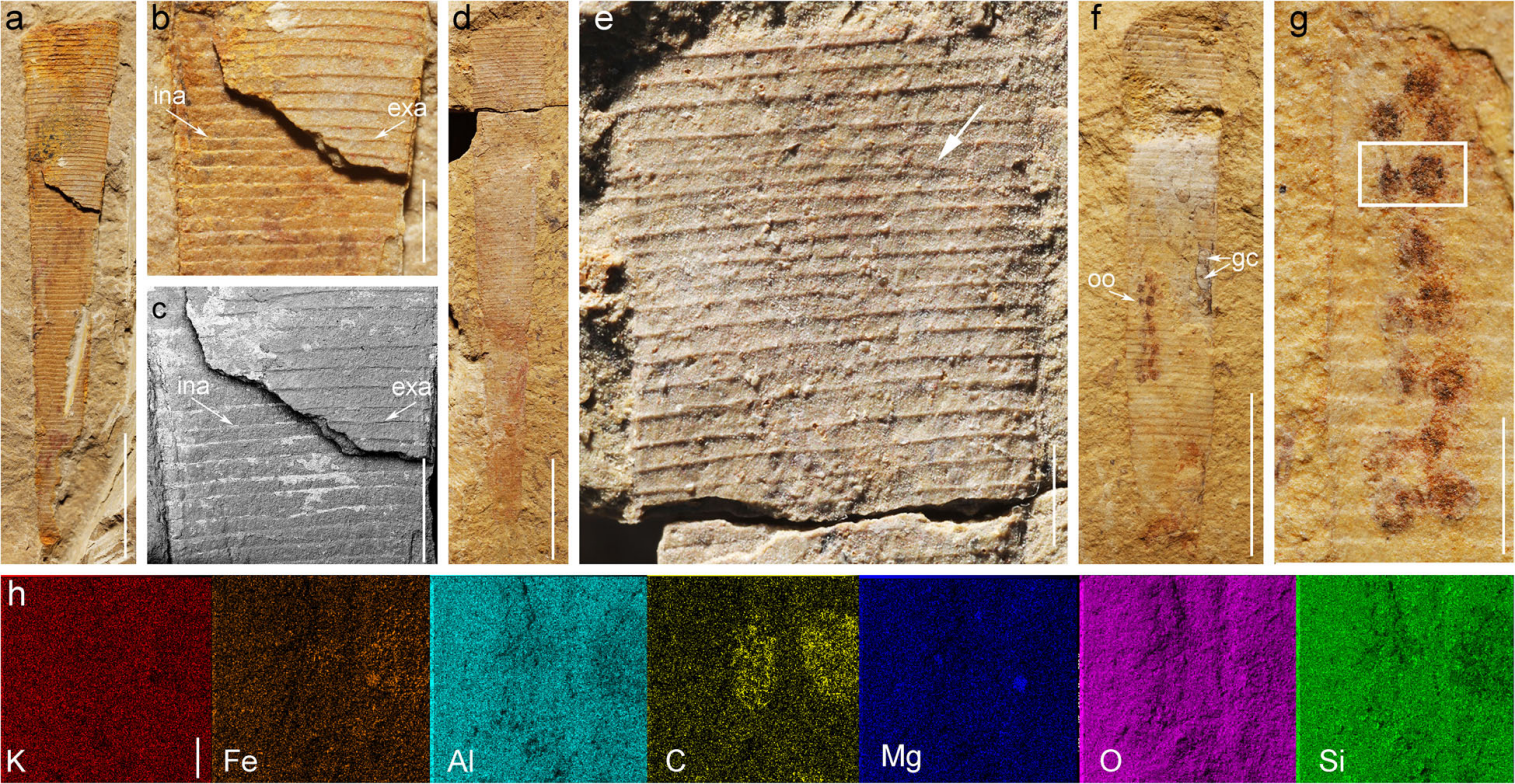
*Selkirkia transita* sp. nov. from the early Cambrian Chengjiang Lagerstätte: tube and eggs. **a**–**c** ELI-0000604, broken tube showing annulations along its external (ridges) and internal surface (furrows). **d**, **e** ELI-0000605, showing annulations (tiny ridges) along the external surface of the tube; general view and close-up (white arrow indicates irregularities in annulated pattern). **f**–**h**, ELI-0000606, showing egg clusters inside the tube; note marked differences with pellet-like gut contents; elemental maps of two eggs (see location in **g**; note enrichment in carbon). Abbreviations: exa, external annulation (ridges); gc, gut contents; ina, internal annulation (furrows); oo, possible ooctyes. Scale bars represent **a**, **d** and **f** 5 mm; **b**, **c**, **e** and **g 1** mm; **h** 100 μm

The present study is based on two species, *Selkirkia sinica* (Figs. 1, 2, 3, 4 and 5) and *S. transita* sp. nov. (Figs. 6 and 7), both from the early Cambrian of China (see below systematic descriptions), that usually occur as isolated specimens and more rarely in relatively large concentration. Clusters of about four specimens (*S. sinica*) per cm^2^ have been found on single bedding planes (e.g. Ercaicun; Additional file 1: Figure S1) and usually show directional polarity, suggesting that individuals were re-oriented by currents. The preservation of *Selkirkia* is identical to that of other associated fossils. Elemental mapping indicates the presence of Al, Si, K, C, Fe, F, P and Mg in the matrix and infilled internal structures (here, intestinal; Figs 1i, 2f and 4g, k, m), suggesting the presence of a magnesium aluminosilicate clay minerals. By contrast, fossil structures reveal concentrations of Fe, as a component of either oxidized pyrite or metamorphic clay phases (Figs. 2f, 3g and 4g, k, m). Traces of C are found on the surface of fossilized tissues (here, gut and oocyte wall; Fig. 2f), as is expected from Burgess Shale-type (BST) preservation [30]. Most anatomical features are underlined by iron oxides which represent the weathered form of original pyrite microcrystals (pseudomorphosis [31]). For example, iron oxides faithfully replicate fine details of the introvert ornament (scalids, teeth) and occur in relatively large abundance over the external surface of the tube and within its wall structure (Fig. 3g).

Numerous fossil specimens show extensive eversion of the whole pharyngeal structure (see also Figs. 1a–c and 2a, d). This extreme state is not seen in living priapulids worms [3, 9] and may result from stress behaviour immediately after death. About 40% to 50% specimens of *S. sinica* and *S. transita* sp. nov. from the Chengjiang Lagerstätte have preserved soft parts. By comparison, *S. columbia* from the Burgess Shale (Canada) is overwhelmingly represented by vacant tubes [4].

### Systematic palaeontology

Phylum Priapulida Delage and Hérouard, 1897

Order Selkirkiimorpha Adrianov & Malakhov, 1995

Family Selkirkiidae Conway Morris, 1977

#### Remark

This family currently accommodates two genera: *Selkirkia* [13] and *Sullulika* from the early Cambrian Sirius Passet Lagerstätte [27]. However, *Sullulika* is an ill-defined genus*,* based on empty annulated tubes comparable with those of Chinese selkirkiids and the supposed presence of convex lappets around the large aperture of the tube [27], that do not occur in any other selkirkiids. The lack of information on its soft anatomy and uncertainties concerning these lappets makes the tentative placement of *Sullulika* within Selkirkiidae uncertain. For these reasons, we find it premature to propose a diagnosis for this family.

*Type genus*: *Selkirkia* Walcott, 1911.

*Genus included*: *Sullulika* [27].

Genus *Selkirkia* Walcott, 1911

### History of research

*Selkirkia* was first erected by Walcott [13] but it was not until 1977 that its anatomy and phylogenetic position were analysed in detail based on the extensive revision of a large number of exceptionally preserved specimens of *S. columbia* from the Burgess Shale [4]. Two decades later, similar fossils were found in the Chengjiang Lagerstätte. Luo and colleagues assigned them to *Selkirkia sinica* [18] and Hou and colleagues to *Paraselkirkia jinningensis* the same year [17]. These tubicolous worms were later discovered in the Xiaoshiba Lagerstätte (Hongjingshao Formation, equivalent of Cambrian Series 2, Stage 3) and assigned to *Selkirkia sinica* [19]. *Paraselkirkia* was erected based on differences with *Selkirkia columbia* from the Burgess Shale (Additional file 1: Table S1), such as the relatively smaller size and more complex introvert structure of *P. jinningensis* [17] (Additional file 1: Text S2) [3, 4]. However, the present systematic revision reveals no major morphological differences between *Selkirkia columbia* and the Chengjiang selkirkiids that would justify maintaining two genera. To us, *Paraselkirkia* is a younger synonym of *Selkirkia* [32, 33] and *S. sinica* and *Selkirkia transita* sp. nov. both belong to *Selkirkia*.

*Type species: Selkirkia columbia* Conway Morris 1977.

*Species included: S. spencei* and *S. willoughbyi* Conway Morris and Robison, 1986, *S. sinica* Luo et al. 1999 and Hou et al. 1999, *S. transita* sp. nov.

*Diagnosis* (emended from Conway Morris, 1977). Selkirkiid worm (body length between 3 mm and 75 mm). Body divided into a spinose introvert and a trunk entirely covered with a tube. Conical introvert with three distinct zones (I to III from proximal to distal). Zone I with two ornamented subzones (Ia and Ic) separated by a smooth ring-like area (Subzone Ib). Fully everted specimens showing more or less developed, smooth area (Zone II) followed by elongated pharynx (Zone III) bearing multispinose or spinose teeth. Finely-annulated tube with low-angle conical shape, and openings at both ends.

*Selkirkia sinica* Lou et al., 1999

1999 *Selkirkia sinica*, Luo et al., p. 81–82, Pl. 20, Figs. 4–6; Text-Fig. 30.

1999 *Paraselkirkia jinningensis*, Hou et al., p. 63–64, Figs. 73–76.

2002 *Selkirkia sinica*, Chen et al., p. 195, Plate 18, Figs. 4-5.

2004 *Paraselkirkia jinningensis*, Hou et al., p. 71, Figs. 12.4–12.5.

2004 *Paraselkirkia sinica*, in Chen 2004., p. 180-181, Figs. 271–272.

2015 *Selkirkia sinica*, Lan et al., p. 125–132, Figs. 1-5.

2017 *Paraselkirkia sinica*, in Hou et al. 2017, p.120–121, Fig. 17.5

2021 *Paraselkirkia sinica*, in Yang et al. 2021, p.3–4, Figs. 1, 2.

*Stratigraphy and locality.* Yu’anshan Formation (equivalent of Cambrian Series 2 Stage 3), *Eoredlichia*-*Wudingaspis* zone, Chengjiang Lagerstätte, Yunnan Province, China; Hongjingshao Formation (equivalent of Cambrian Series 2 Stage 3), *Yunnanocephalus*–*Chengjiangaspis-Hongshiyanaspis* zone, Xiaoshiba Lagerstätte, Yunnan Province, China.

*Diagnosis* (emended from Luo et al., 1999 and Hou et al., 1999). *Selkirkia* with a relatively small size (< 20 mm long on average). Subzone Ia with spines arranged in irregular quincunxes. Subzone Ib well-developed. Subzone Ic with spines arranged in dense and regular quincunxes. Pharynx bearing numerous tiny, evenly spaced spinose teeth. Trunk possibly extending posteriorly into two lobe-like caudal appendages. Oocytes are concentrated within the posterior half of the body cavity on either side of the gut. Tube bearing evenly spaced annulations (8 to 14 per mm).

### Description

#### Introvert

Fully everted introverts show three distinct spinose (Ia, Ic and III) and two smooth areas (Ib and II) (Figs. 1a, b and 2a). The length of the best-preserved fully everted introvert is about 25% of total body length. Zone I is subdivided into three subzones termed Ia, Ib and Ic from proximal to distal. The boundary between the Zone I and Zone III (pharynx) is marked by a smooth, slightly constricted area (Zone II).

*Subzone Ia* (Figs. 1a, b and 2a). Its diameter is equal or slightly smaller than that of the anterior opening of the tube from which it protrudes. Its numerous stout spines are distributed in discrete quincunx, directed backwards and decrease in size posteriorly. They form six circles and 12~13 longitudinal rows in one side.

*Subzone Ib* (Figs. 1a, b and 2a). This smooth area marks the boundary between Subzone Ia and Subzone Ic. Its length is one-third to one-fourth that of Subzone Ia.

*Subzone Ic* (Figs. 1a, b and 2a). Characterized by relatively closely spaced, slender and long spines (*N*=ca 250) arranged in quincunx and directed backwards.

*Zone II* (Figs. 1a, b and 2a): This smooth area lies between Zone I and Zone III.

*Zone III* (Figs. 1a, b and 2a). This ornamented zone represents the pharynx. Its morphology and length vary depending on the degree of eversion (Figs. 1a–c and 2a, d). In fully everted specimens, the pharynx appears as a slender conical structure bearing a large number (*N* > 200) of tiny teeth, all of virtually the same size (Additional file 1: Fig. S2b). They arrange quincunxially as seen in the best-preserved specimens. Their morphology is simple (Additional file 1: Fig. S2c, f) compared with that of the multi-cuspidate teeth seen in *S. columbia* ([4, 21]; Additional file 1: Fig. S2i, j). Some of our specimens show a bulbous structure at the distal end of the pharynx (Figs. 1a and 2a).

#### Trunk

A single specimen shows the body of the animal almost completely pulled out from its tube (Fig. 5o, p). The body is almost as long as the tube. Although faint traces of the gut are discernible, the external boundary of the trunk remains relatively featureless but with weak annulations (Additional file 1: Fig. S3b, c, e, f). It does not seem to have suffered from severe decay as indicated by its consistent cylindrical shape and a relatively well-preserved introvert. In other specimens, the distal part of the trunk protrudes outside the large opening of the tube in different lengths allowing the trunk cuticle to be observed (Figs. 1a, 2a, 3e, 4a and 5; Additional file 1: Fig. S3). It is represented by a 20~30 μm thick reddish layer (Figs. 3f and 5b, d, l, n) approximately one third of the tube wall (ca. 100 μm; measured in empty tubes; Fig. 3b, d, g). Small, irregularly spaced protuberances are present around the protruding trunk (Fig. 5f) and recall the papillae seen in the middle part of the trunk of *S. columbia* [4]. However, they do not distribute in rows and may not have a biological origin. Some specimens show a tiny gap between the tube and the external surface of the trunk (Fig. 5d, f, h, k), which suggests that the body was free from the tube and could possibly move within it. In the vast majority of individuals, the trunk is some distance away from the internal surface of the tube (e.g. posteriorly) although in contact with it locally (Fig. 1d, e). The exact morphology of the trunk end is unclear but may have born a pair of relatively short caudal appendages that can be seen protruding outside the small opening of the tube (Fig. 1g, h) or in a more retracted position (Fig. 1e, f).

#### Gut

The gut generally appears as a dark strip running from the everted pharynx to the trunk end (Figs. 1, 2, 3 and 4). The anus seems to open close to the posterior opening of the tube (Fig. 1f) and within the axial plane of the animal. The gut has an axial position and a straight outline although loops can be seen in some specimens (Fig. 2a, b). It is often filled with pellet-like elements aligned in rows or more irregularly distributed (Fig. 2c–e). These gut contents have a consistent ovoid shape (0.5~0.6 mm and 0.15–0.20 mm in length and width, respectively) and present a slight relief. Their relatively large number and local concentration suggest that the gut wall had the capacity to expand in order to accommodate food or undigested residues [19, 34]. Scanning electron microscope (SEM) observations revealed no internal elements that may help characterize the worm diet. Two ovoid features of equal size occur near the posterior end of one specimen (Fig. 2c) and seem to be aligned within the gut tract. However, their size (1.5 mm long, 0.9 mm wide) is larger than that of the pellet-like elements usually found in a more anterior location within the gut. Their width is almost three times the gut diameter (0.5 mm in width). They may represent undigested shells (e.g. brachiopod), or unusually large pellets transiting through the digestive tract (Fig. 2e). Possible faeces seen in a few specimens consist of shapeless coloured clusters of minerals such as mica and quartz grains (Fig. 4n, o).

#### Tube

The tube forms a conical structure with a very low opening angle (ca 20°) and opens at both ends. Its length ranges from 4 to 16 mm with the proximal opening being 1 to 2 mm wide (*N* = 120, see Additional file 2: Excel S1). The external surface of the tube is regularly annulated with 8 to 14 tiny transverse ridges per millimetre (Fig. 1b) that faithfully correspond to comparable small furrows along the internal surface. The tube wall (ca. 100 μm; Fig. 3b, d, g) is preserved in iron oxide (Fig. 3g) and does not show any ultrastructural details (e.g. cuticle layers). The posterior opening of the tube is relatively narrow and often broken (irregular margins) due to possible interactions with sediment or transportation (Fig. 4a). Tiny ovoid, relatively featureless objects (size between 1 and 1.5 mm; Fig. 4b–n) occur in about 810 specimens (ca. 45%), that seem to be attached to the external wall of the tube, close to its posterior end. A comparable association recently described in *Selkirkia* from the slightly younger Xiaoshiba Lagerstätte [35], provides clear evidence that these rounded objects are actually brachiopod epibionts.

#### Remarks

Our specimens do not show noticeable differences with the type specimens of *S. sinica* figured by Luo et al. (1999; plate 20, text-figs 4–6) and Hou et al. (1999; text-figs 73–76) and those more recently described from the Xiaoshiba Lagerstätte [19]. All display the same type of everted spinose introvert divided into two parts and elongated pharynx bearing teeth. Detailed observations of the arrangement, density and morphology of cuticular elements (scalids, pharyngeal teeth) did not reveal small-scale differences either (Additional file 1: Table S1).

*Selkirkia transita* sp. nov.

*Etymology.* From *transita* (Latin), alluding to resemblances with both *S. sinica* and *S. columbia*.

*Holotype*: ELI-0000601 (Fig. 6a)

*Paratype*: ELI-0000602, ELI-0000603 (Fig. 6f, i)

*Stratigraphy and locality.* Yu’anshan Formation (equivalent of Cambrian Series 2 Stage 3), *Eoredlichia-Wudingaspis* zone, Chengjiang Lagerstätte, Yunnan Province, China.

*Diagnosis. Selkirkia* with a relatively large size (> 20 mm on average). Subzone Ia with spines arranged in discrete quincunxes. Subzone Ib very narrow to virtually invisible. Subzone Ic with spines in dense and regular quincunxes. Pharynx bearing hundreds of multispinose teeth arranged quincunxially, pointing forwards and decreasing in size distally. Oocytes in the posterior half of the body cavity and possibly arranged in two longitudinal rows. Tube bearing evenly spaced external annulations (5 to 9 per mm).

### Description

#### Introvert

*Subzone Ia* (Fig. 6a–d). It is the widest part of introvert. Spines distribute in discrete quincunxes with 6 circlets of about 25 spines arranged longitudinally. The distance between adjacent spines in diagonal is about 0.15 mm. Spines increase in size from proximal to distal part. The shortest and longest spines are 0.12 mm and 0.27 mm long (basis 0.10 mm and 0.12 mm), respectively.

*Subzone Ib* (Fig. 6d). Relatively narrow (ca 0.2 mm in longitudinal length), smooth and marks the boundary between Subzone Ia and Subzone Ic.

*Subzone Ic* (Fig. 6d). Slightly narrower than Subzone Ia and varies in length from 1.0 mm to 2.3 mm. Subzone Ic bears closely packed spines arranged in quincunx. Spines increase in size gradually from proximal to distal (the shortest and longest ones are 0.10 mm and 0.34 mm, respectively) and all point backwards. The basal width of spines is about 0.07 mm. Distal spines are three times longer than proximal ones.

*Zone II* (Fig. 6e). In specimens with a fully everted introvert, its length is 25% that of Subzone Ic and its diameter decreases distally.

*Zone III* (Fig. 6e). Corresponds to the pharynx and has a tapering shape especially well-marked in its terminal region. Bears numerous teeth arranged quincunxially and regularly decreasing in size distally. The most proximal ones (basal width between 0.05 and 0.11 mm) are clearly multispinose with one long central tip flanked with two smaller ones (Fig. 6e; Additional file 1: Fig. S2d, e, g). The remaining spines appear as densely coloured dots of ca 0.02 mm in diameter with no visible details and point forwards.

#### Trunk

In one specimen, the broken part of the tube reveals details of the relation between trunk and tube (Fig. 6f) with a gap between the cuticle and the internal surface of the tube (Fig. 6f). Possibly paired retractor muscles (Fig. 6h) seem to be attached to the middle part of the trunk.

#### Tube

The conical tube of *Selkirkia transita* sp. nov. is 10 to 38 mm long (see Additional file 2: Excel S1) and opens at both ends (Fig. 7a, d). As in *Selkirkia sinica*, it bears tiny low-elevated transverse ridges along its external surface that correspond to furrows along its internal wall (Fig. 7a–c). Most ridges are regularly spaced (commonly 5 per mm) although local variations (from 5 to 9 per mm) may occur. Some ridges seem to fuse or bifurcate (Fig. 7d, e). Although often broken and damaged the posterior end of the tube seems to have a sharp ovoid opening with no additional cuticular features.

#### Gut

As seen in *S. sinica*, it appears as a narrow tube running from the tip of introvert to the anus and often contains pellets (Fig. 6f).

#### Reproductive system

Clusters of spherical elements (Nmax = 14, diameter between 270 and 480 μm) found in the posterior part of the body cavity of *Selkirkia transita* sp. nov. and are highlighted by reddish iron oxides and small black patches (Fig. 7f, g). They form either a single cluster close to the inner wall of the body or irregular paired longitudinal rows on either side of the gut (Fig. 7g). Similar features (less than 30; diameter between 300 and 450 μm) occur in a few specimens of *Selkirkia sinica* from the Xiaoshiba Lagerstätte and were recently interpreted as oocytes based on comparisons with extant priapulid worms [35].

#### Remarks

*S. transita* sp. nov. has an introvert and a stiff tube, that closely resemble those of *Selkirkia columbia* [4] but shows differences with other *Selkirkia* formerly described in the literature. In general, the size of *S. transita* sp. nov. is larger than that of *S. sinica* but smaller than that of *S. columbia* [4, 17, 18]. Subzone Ib of the introvert is poorly developed in *S. transita* sp. nov., absent in *S. columbia* and instead well-marked in *S. sinica*. The most anterior spines of Subzone Ic point either outwards or backwards in *S. transita* sp. nov. and *S. sinica*, but always forwards in *S. columbia*. Concerning the pharyngeal region, only one type of multispinose teeth part, is present in *S. transita* sp. nov., whereas two types occur in *S. columbia* [21]. In contrast, *S. sinica* seems only to bear simple teeth. The tube bears about 40 annulations per mm in *S. columbia*, 8 to 14 in *S. sinica* and only 5 to 9 in *S. transita* sp. nov. The morphological differences between *S. sinica* and *S. transita* sp. nov. were all observed in specimens of approximately the same size, which rules out the possibility that both forms represent ontogenetic stages of a single species. Altogether, this whole set of morphological divergence justifies the erection of a new species (Additional file 1: Table S1).

### Relation of tube to body

In both species, the tube wall (ca. 100 μm; Fig. 3b, d, g) is preserved in iron oxide (Fig. 3g) and does not reveal any ultrastructural details (e.g. cuticle layers). It is about three times thicker than the cuticle that covers other parts of the worm’s body (ca. 20 to 30 μm; Fig. 3f). The introvert is frequently the only part of the worm that protrudes outside the anterior opening of the tube and displays varying degrees of extension (from contracted to fully everted state showing pharynx). However, in numerous specimens a substantial portion of the trunk stands exposed outside the trunk (Figs. 1a, 2a, 3e, 4a and 5; Additional file 1: Fig. S3) suggesting that the worm could move freely within its tube. The lack of a large gap between the trunk and the inner surface of the tube indicates that the worm could probably slide along its protective encasement.

In one specimen of *S. sinica* the worm’s body is almost completely pulled out from its tube (Fig. 5o, p). Although extremely slim, its trunk displays regular outlines. This configuration may represent the worm in the process of extricating itself from the tube (Fig. 5o, p).

### Phylogenetic relation of *Selkirkia* to other scalidophoran worms

Our phylogenetic results differ considerably from those that were published before [10], owing to our extensive recoding and implementation of neomorphic/sovereign characters (see the ‘Methods’ section).

Heuristic search with Tree bisection and reconnection (TBR) only and Tree with new technology analyses (TNT) retrieve topologies with basal Loricifera and Kinorhyncha forming a sister clade to other scalidophorans, most fossils being part of the priapulid stem group, although internal branching is mostly polytomous (Additional file 1: Fig. S4a, b). By contrast, the unconstrained Bayesian runs converge on a basal *Selkirkia* sister group to Palaeoscolecida and Scalidophora (Additional file 1: Fig. S5). In the unweighted TreeSearch analysis (Additional file 1: Fig. S4c), Loricifera and Kinorhyncha are also resolved basally, but *Selkirkia* is retrieved in the closest sister clade to crown Priapulida, along an extensive fossil priapulid stem. When using implied weighting in TreeSearch, consensus trees for individual concavity constants are well resolved, but they significantly differ from one another, leading to a polytomy if one combines these results (Additional file 1: Fig. S4d). These topological discrepancies between concavity constants [36, 37] constitute an example that the method of implied weighting may indeed be too inconsistent despite good performances with simulated data [38].

**Fig. 8:**
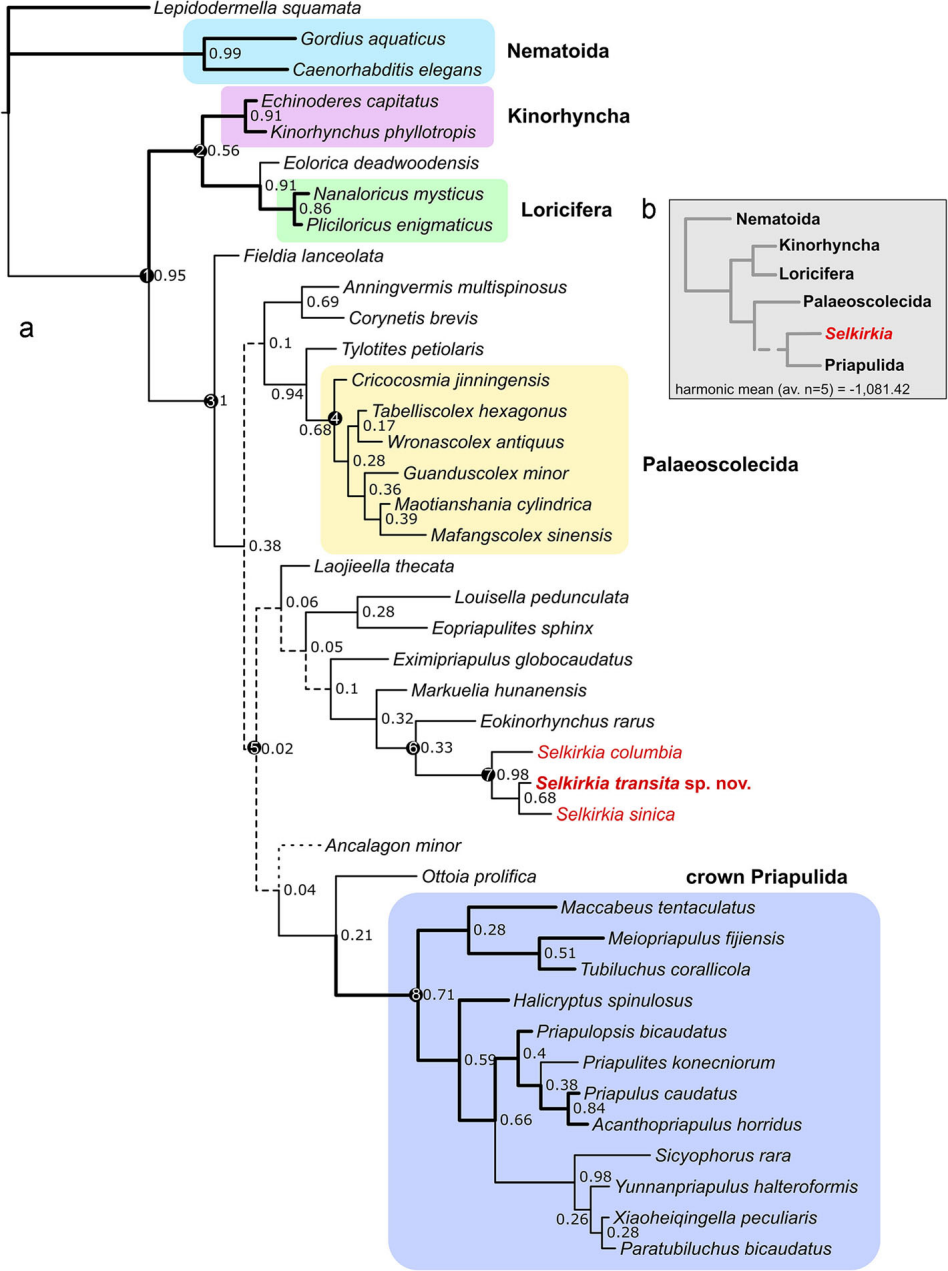
Maximum clade compatibility tree from a Bayesian analysis using an Mkv+Γ model and a backbone enforcing basal Kinorhyncha and Loricifera. **a** Full tree. Bold branches lead to extant lineages. Numbers next to node are posterior probabilities. Circled numbers at nodes are key apomorphic characters: 1, eversible introvert in adult. 2, retractable mouth cone and oral elements. 3, eversible pharynx lined with teeth in multiple rows. 4, scalids distributed only at anterior part of Zone I, one to several rows of plates in each annulation, paired anal hooks or setae. 5. twenty-five longitudinal rows of scalids arranged quincunxially (Additional file 1: Fig. S7. This trait may be shared convergently by priapulids and *Selkirkia*) and terminal anus. 6, multi-subdivisions Zone I. 7. cuticular and annulated tube. 8. The arrangement of crown priapulid scalids shows 8+9+(8+8+9) pattern with the exception of fossil taxa. **b** Simplified topology showing the position of *Selkirkia* and harmonic mean (−1081.42). Dashed lines mark branches supported by extremely low posterior probabilities (≤0.1). The dotted line denotes the instability of *Ancalagon minor*

A basal position of Loricifera and Kinorhyncha with a large total-group Priapulida being consistently recovered by parsimony analyses, we decided to test the strength of this model by enforcing a backbone on the Bayesian analysis. The resulting topology (Fig. 8) bears overall similarity with the unweighted TreeSearch topology in the basal placement of palaeoscolescids and yields on average slightly better harmonic means than the unconstrained model (− 1083.58 vs. − 1081.42, averaged each 5 searches; see Additional file 3: Excel S2). A better treatment of inapplicable states in TreeSearch therefore helped parsimony converge with Bayesian likelihood, assuming a model with basal Loricifera and Kinorhyncha. However, major scalidophoran nodes have abysmal posterior probabilities of 0.1 or below in the likelihood analysis (Fig. 8; Additional file 1: Fig. S5). We therefore consider the constrained Bayesian tree as the representative of the best evolutionary model for our data, but the stability of this model, and in particular the basal placement of palaeoscolecids in total-group Priapulida, will require further testing (Fig. 8). *Selkirkia* is otherwise consistently retrieved in both Bayesian analyses in a clade with *Markuelia* and *Eokinorhynchus*, suggesting an order-level grouping defined by the subdivisions of the Zone I of the introvert.

*Selkirkia* has long been assigned to priapulids based on overall similarities with extant priapulids, such as the introvert structure, pharyngeal teeth and trunk annulation [4, 11, 18, 39–43]. Other authors have considered the tube of *Selkirkia* has the equivalent and a possible homologue of the lorica of extant priapulid larvae and loriciferans [44], and advocated a position within scalidophorans without specifying to which group it may belong [28, 29, 44–46]. *Selkirkia* clearly differs from extant Kinorhyncha and Loricifera, which both are characterized by the presence of a mouth cone and oral stylets [47]. There are no such oral features in *Selkirkia* which instead has multispinose pharyngeal teeth arranged quincunxially as in numerous fossil and extant priapulid worms. Another major difference with these two groups is that the trunk of *Selkirkia* bears no external “segments” (the so-called zonites of kinorhynchs). Its finely annulated tube most probably secreted by the epidermal cells of the trunk (see below) has no equivalent in other groups.

Our favoured phylogenetic result (Fig. 8) implies that the *Selkirkia* clade (with *Laojieela* as its most basal member) might be part of the close stem of Priapulida, and could therefore document a morpho-anatomy lying close to the priapulid radiation. The priapulid affinities of *Selkirkia* are supported by the following set of morphological characters: (1) twenty-five longitudinal rows of scalids are found in *Selkirkia* and the vast majority of extant priapulids (except *Meiopriapulus fijiensis*); (2) the pharynx of *Selkirkia* is lined with multispinose teeth as in extant [26] and Cambrian [4] priapulids; and (3) the homology between the tube rings and the cuticular annulations seen in modern priapulids. However, the first trait is absent in most basal members of the putative *Selkirkia* clade (e.g. *Eopriapulites*, *Laojieella*) as well as *Ottoia* (Fig. 8), which would imply either broad convergences or that *Selkirkia* may lie even closer to crown priapulids.

In addition, the introvert of *Selkirkia* is subdivided in the same way as that of extant priapulids (i.e. Zone I, II, III) and clearly differs from that of kinorhynchs and loriciferans (Zone I, Zone II, and mouth cone). The possible paired caudal appendages of *Selkirkia* have counterparts in some extant (e.g. *Priapulus* [26]) and fossil (e.g. *Paratubiluchus* and see [26, 48]) priapulids.

In this scenario, the crown of Priapulida includes the Carboniferous *Priapulites*, the early Cambrian *Paratubiluchus*, *Xiaoheiqingella, Sicyophorus*, and *Yunnanpriapulus*. *Ottoia* and possibly *Ancalagon* are the closest priapulid stem taxa. *Fieldia* is here resolved at the basalmost position of total-group Priapulida.

Considering the weak support of higher nodes, the alternative topology based on an unconstrained Bayesian analysis which retrieves *Selkirkia* and palaeoscolecids as stem scalidophorans (Additional file 1: Fig. S5) should not be too hastily discarded. This scenario implies that many important priapulid apomorphies—as evidenced by *Selkirkia*—appeared very early in the evolution of the group, with kinorhynchs and loriciferans secondarily losing some of these traits (e.g. 25 longitudinal rows of scalids). This contrasts with the topology described above according to which the known Cambrian fossil radiation was mostly linked to the build-up of the priapulid body plan.

## Discussion

### Tube formation: agglutination and accretion hypotheses

The tube of *Selkirkia* has been subject to various interpretations. It is considered by some authors [4] as a cuticular structure separated from the trunk by a gap, suggesting that the animal was free to move within its tube. Based on comparisons with *Maccabeus* Conway Morris [4] hypothesized that the tube of *S. columbia* was secreted by substances possibly emitted from hooks present around the proximal introvert (Subzone Ia). However, there is neither solid evidence of such specialized hooks in *S. columbia* [4] (Additional file 1: Fig. S2h) and the congeneric Chinese species [22] (Figs. 1 and 6) nor visible organs along its introvert and trunk that might indicate any excretion process as suggested by Conway Morris [4]. *Maccabeus tentaculatus* [49] is a less than three-mm-long priapulid that lives within a cylindrical thin and flimsy tube open at both ends, that is apparently formed via the agglutination of plant fragments (e.g. *Posidonia*) by sticky substances emitted through glandular spines [49]. The tube of *Selkirkia* neither has exogenous organic elements nor sediment particles attached to its internal or external surface (Fig. 7b, c, f) and is therefore unlikely to result from a comparable construction mode. Some extant annelids secrete conical structures (e.g. calcareous and particulate tube of *Ditrupa* (Serpulidae) and *Pectinaria*, respectively [2]). However, their construction proceeds by accretion and requires a specific organ (collar, behind the prostomium) that does not exist in any extant scalidophoran worm. The extremely regular annulated ornament of *Selkirkia* that occurs on both sides of its tube (positive and negative along external and internal wall, respectively) is also poorly consistent with such an accretional process. The external corrugations or collared morphologies (e.g. siboglinids [2]) of modern annelids have no equivalent in *Selkirkia*.

### Tube formation: the lorica hypothesis

Some authors (e.g. refs. [28, 29]) have suggested that the tube of *Selkirkia* was an integral part of the trunk cuticle and that body and tube were inseparable elements. In this case, the tube would be nothing more than a cuticular thickening formed around the trunk, by the addition of chitin layers and would have a direct equivalent in the lorica of extant and fossil scalidophorans. (e.g. *Priapulus*; see [23]).

Detailed studies on the early ontogeny of priapulid worms (e.g. *Priapulus*; see [23]) show that, contrary to loriciferans, priapulid hatchlings lack a lorica. The hatching larva then gives rise to at least two successive loricate larval stages. These larvae have a functional introvert, a neck and a trunk encased within a protective vase-like structure (lorica) made of 8 longitudinal cuticular plates. The second loricate larva is characterized by the opening of the mouth and anus. The next developmental stage discards the lorica during moulting and resembles adults in having a vermiform and flexible body lined with a thin cuticle. In contrast with priapulids, loriciferans maintain a lorica throughout their lifecycle [3]. Loriciferans are tiny interstitial animals of a few hundred microns long, characterized by a unique specialized anatomy (e.g. scalids) and life history [3].

Although the tube of *Selkirkia* is also arguably secreted by epidermal cells, it differs from loricae in various aspects. First, *Selkirkia*’s tube is eventually detached from the trunk, probably as part of ecdysis. The lorica is likewise renewed by moulting but remains always tightly attached to the body during the inter-moult stages, whereas the tube of *Selkirkia* would go through a binary cycle of continuous growth followed by detachment from the body and thus growth stagnation (Additional file 2: Excel S1). Second, while the lorica of some loriciferans species such as *Rugiloricus ornatus* seem to have both longitudinal and transverse folds [50], most extant and fossil loricae [3, 6, 7, 18, 51] are characterized by the presence of longitudinal ridges. Moreover, the tube of *Selkirkia* is sclerotized and rigid contrasting with the flexibility of extant loricae (e.g. flexible strips between plates [52]).

Because a larval lorica is known in loriciferans and priapulids but unknown in fossil scalidophorans, with the exception of *Sicyophorus* [18] and *Eolorica* [7], the presence of a loricate larva could be optimized as plesiomorphic for Scalidophora in our favoured topology (Fig. 8). This would imply that the tube of *Selkirkia* could represent an extensively modified lorica, driven by protective needs. However, the hatching larvae of priapulids lack a lorica, questioning the homology of the lorica between priapulids and loriciferans. The fossil *Markuelia*, resolved close to *Selkirkia*, also arguably lacks a lorica [53]. In light of this and considering the remarkable differences in construction mode and renewal between loricae and the tube of *Selkirkia*, with no fossil evidence currently shedding light on the early developmental stages of *Selkirkia*, the developmental origin of its tube cannot be ascertained.

In summary, regardless of deep developmental origins, the tube of *Selkirkia* cannot be regarded as a lorica *sensu stricto* and is arguably not a structure formed by the agglutination of exogenous particles. Instead, it is more likely to constitute a sclerotized feature that was repeatedly shed by ecdysis and renewed and played a protective role throughout the life cycle of the animal except during the short period needed for its secretion.

### Moulting in *Selkirkia*

**Fig. 9:**
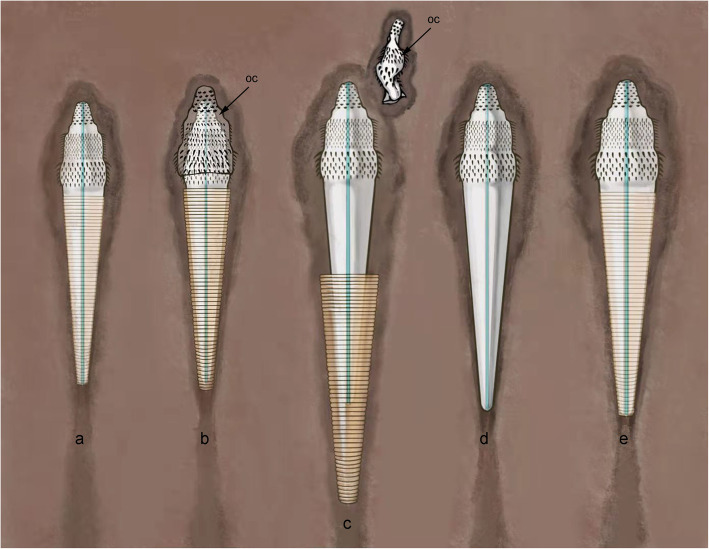
Sequence diagram to explain the tube formation and moulting in *Selkirkia* (Cambrian). **a** Worm attached to its body (just before moulting). **b** Ecdysis: freshly moulted worm splits from its old cuticle (black arrow) (tube + thinner cuticle all over the body). At that stage, there is a gap between the worm and its tube. The worm can move within it. **c** Worm grows in size within its tube, mainly in length, allowing the body including trunk to protrude outside. **d** Worms leaves and discards its old protective tube. At that stage, it is well protected by a new cuticle but the tube has not formed yet. **e** Worm secretes a new tube around its trunk as in **a**. Abbreviation: oc, old cuticle. Note that *Selkirkia* is seen from above, and although *Selkirkia* was mainly epibenthic, moulting may have occurred slightly below the water/sediment interface

We propose the following scenario to explain how the tube of *Selkirkia* was formed and renewed and more generally how the whole moulting process took place. This scenario implies that the body of the worm stands free of its tube at some stage and could move within it. (Fig. 9): (1) The epithelial cells of the trunk secreted a cuticular layer that grew in thickness to eventually form a rigid tube. Sclerotization did not occur elsewhere (e.g. the introvert was lined with a thin and flexible cuticular layer). At this stage, the future tube grew in size along with the rest of the animal. (2) Ecdysis took place after the tube construction was completed, which resulted in splitting the freshly moulted worm from the inner wall of the tube, thus creating a narrow gap between the tube and the body wall. At that stage, the whole worm was already covered and protected by a flexible cuticular layer as in all non-tubicolous ecdysozoans, and experienced a surge in mass and length increase. (3) The worm’s body mass kept increasing (as is characteristic of all ecdysozoans) and could move freely within it. At that stage, part of the trunk was able to protrude outside the anterior opening of the tube. Circular, longitudinal and retractor muscles allowed the worm to slide within its tube and outside by exerting local pressure on the hydrostatic skeleton [54]. (4) After optimal body growth was achieved, the worm left and discarded its tube.

### Locomotion of *Selkirkia*

We suggest that locomotion was achieved via the eversion and anchoring of introvert (scalids) to sediment that allowed the worm (when attached to its tube; see stage in Fig. 9a) to drag its tube and move forwards. Clearly, the tube must have represented an extra-weight for the animal thus hindering or limiting its capacities to move through its environment, compared with non-tubicolous worms. After splitting from its tube via ecdysis (stages in Fig. 9b, c), the worm could move within its tube but the lack of extensive contact between body and tube at that stage might have made displacements more difficult. It is only after leaving its tube that the worm could probably move the fastest through its environment, by using the same dynamic system that characterizes non-tubicolous worms (longitudinal and circular muscles transmitting pressure to the fluid-filled body cavity (hydrostatic skeleton) allowing all body parts to change in shape and stiffen). In summary, *Selkirkia* is seen as a slow-moving, possibly semi-sessile animal during a major part of its lifecycle.

### Commensalism

Tiny brachiopods attached to the tube of *Selkirkia sinica* were recently described from the Xiaoshiba Lagerstätte (ca. 514 Ma) [35]. They resemble tiny brachiopods epibionts (Fig. 4b–n) such as *Inquilinus* [55] and *Kuangshanotreta* [56] that are found attached to various elements of the Chengjiang biota, such the larger brachiopods *Diandongia* [55], algae-like organisms such as *Malongitubus* [56], and the scalidophoran *Cricocosmi a*[57] (Additional file 1: Fig. S6). It makes little doubt that the same association also occur in the slightly older Chengjiang Lagerstätte (Fig. 4b–n) although the specimens from this locality are less well preserved than those from Xiaoshiba Lagerstätte [35]. Some discussion is necessary concerning this unusual association, especially whether the brachiopod was attached to the worm [35] and not the other way round.

Attaching to a hard substrate (e.g. skeletal element) makes sense if it provides stability to the animal. Numerous epibionts (including brachiopods) settle on shell fragments and anchor to it via their pedicle, thus avoiding sinking into soft sediment (see [54]). It seems unlikely that brachiopods of less than 1.5 mm could have offered any stable anchoring to *Selkirkia*. Interestingly, some extant loriciferans glue themselves to sand grains by secreting sticky substances (see [58]). If we suppose that *Selkirkia* had a similar behaviour, then diverse elements would have been found scattered over the tube, which is not the case. A more localized sticking spot (e.g. via substances emitted from the posterior opening) would not explain either why *Selkirkia* settles on tiny brachiopods rather than other biological or inorganic elements abundantly represented at the water/sediment interface.

In summary, the hypothesis of brachiopod epibionts seems to be the most plausible one. There remains the puzzling question of the exclusive location of these brachiopods, close to the posterior end of the tube (Fig. 4; see also [35]).

### Habitat and tubicolous lifestyle

The brachiopod feeding mode is generated by lophophoral cilia and requires constant contact with circulating water [2]. These ecological constraints and the consistent location of brachiopods near the posterior end of the worm tube would suggest that *Selkirkia* probably lived at the water/sediment interface, possibly lying in a subhorizontal or slightly tilted position, although very shallow incursions into the sediment should not be excluded (see below).

The assumed moulting process of *Selkirkia* implies that the worm temporarily left its tube and probably remained buried in sediment until the renewal of its tube. Although not strictly sedentary, *Selkirkia* most probably had limited capacities for colonizing distant areas compared to other worms (see above). This would have resulted in gregarious habits as suggested by relatively dense concentrations of individuals found in some localities (Additional file 1: Fig. S1). The pharyngeal teeth of *Selkirkia* suggest a feeding mechanism comparable with that of modern priapulid worms, i.e. tiny prey of detritus grasped by the everted pharynx and drawn into the gut (Additional file 1: Fig. S2, [9]). Pellet-like elements found within its digestive tract (Fig. 2c–f) would indicate that *Selkirkia* ingested a substantial amount of sediment mixed with food (possible meiobenthic prey or organic detritus).

### Tubicolous habits in early animals

Tubular exoskeletal features are frequent and diverse in numerous present-day animals (e.g. cerianthid cnidarians, annelids, pterobranch hemichordates). This strategy occurs in metazoans as old as the late Ediacaran (e.g. cloudinomorphs [59]), and the lowermost Cambrian (e.g. Kuanchuanpu Formation, ca. 535 Ma; *Cloudina*-like animal, [60]); however, the exact nature of these first tubicolous organisms and how they secreted and built their tube has not yet been elucidated.

Tubicolous bilaterians have also been recently described from various Cambrian Lagerstätten [61–64]: (1) *Facivermis yunnanicus* is an atypical lobopodian (Panarthropoda) [61, 65], with an elongated vermiform body lacking posterior appendages, and is also supposed to have secreted a stiff protective tube. We concur with Howard et al. [61] that the nature of its tube remains uncertain. Its strong pyritization would rather support a cuticular structure tightly associated with the body, than one built from sediment (e.g. via mucous secretions). (2) *Dannychaeta tucolus* is an early Cambrian annelid (Stage 3, China) that is also assumed to have been living in a relatively spacious tube [62], approximately four times the body width. However, it is unclear whether this tubular structure, mineralized in pyrite, represents a constructed organic tube or simply a consolidated burrow coated with mucus. (3) *Spartobranchus tenuis* from the mid-Cambrian Burgess Shale is a hemichordate that secreted a corrugated tube [63] inside which the animal could move freely. Some of these tubes show bifurcating features. (4) Similarly, the other Burgess Shale hemichordate *Oesia disjuncta* [64] lived in filamentous, probably collagenous bifurcating tubular structures formerly described as the “alga” *Margaretia dorus* [66]. The tube of *Oesia* was compared to that of extant colonial hemichordates, although it is less clear how such complex structures were constructed by fully-grown individuals. Latest evidence revealed that the tube of *S. tenuis* may be a pseudo-colonial structure built collectively by hemichordate zooids [67].

Tubular lifestyles have therefore appeared independently at the earliest stages of animal evolution in groups as diverse as hemichordates, annelids, lobopodians and, as shown in this study, scalidophorans. Such a spectacular case of convergence—or parallelism, in cases where common genes were co-opted in the tube-building process [68]—may have resulted from the combination of a labile vermiform body plan, observed across the stem of several animal phyla, as well as the arguably higher morphological variability of many extinct forms during the Cambrian, characterized by a weaker canalization of genotypic-phenotypic pathways [69, 70]. Considering that the tubes had the advantage of better protecting the soft body of these vermiform organisms from physical damage, this convergent evolutionary trait may be seen as a response to the high environmental and biological pressure that accompanied the early radiation of animals and the construction of richer marine ecosystems.

By contrast, most extant enteropneust hemichordates are crawlers and burrowers and the vast majority of scalidophorans, overwhelmingly represented by priapulids, are active infaunal non-tubicolous organisms that spend most of their life cycle buried in sediment. The advantages of tube-dwelling, such as physical self-protection and stronger anchoring to sediment, are counterbalanced by a reduction of the animal's ability to move through its environment as we have seen in *Selkirkia*, which may have had consequences for its survival (e.g. access to food sources, dispersal, reproduction). It seems therefore that mobility prevailed in these groups on the long term. Consequently, the selection of either protection or mobility may be seen an important evolutionary trade-off that controlled the development of tube-dwelling since the early stages of animal life.

## Conclusions

*Selkirkia* is an example of a scalidophoran worm living in a tube and has no direct equivalent among fossil and extant Ecdysozoa. The tube is a cuticular conical structure secreted by the epithelium of the trunk and likely renewed through ecdysis as the rest of worm’s cuticle. In contrast to non-tubicolous worms, *Selkirkia* was most probably a semi-sessile organism, able to move within tube during part of its life cycle, possibly to feed near the water-sediment interface. Although a deep developmental origin remains possible, the tube thus clearly differs from the lorica of fossil and extant scalidophorans (e.g. priapulids, loriciferans) which remains inseparable from the worm’s body, suggesting that various cuticular protective structures have evolved independently in various groups of scalidophorans. The annulated tube itself may have played an important role for protection and anchoring in sediment. It also served as a host substrate to tiny brachiopod epibionts, suggesting that *Selkirkia* was not an infaunal worm as the vast majority of coeval scalidophoran worms. Our critical phylogenetic re-evaluation emphasizes the existence of a conflict between the placement of the majority of Cambrian fossils as either a long stem to Priapulida or to Scalidophora, but our model-based approach provides an objective means of assessment and should be used more systematically in fossil-based cladistic studies.

## Methods

### Materials

Our fossil specimens (*Selkirkia sinica* and *Selkirkia transita* sp. nov.) come from several localities of the Chengjiang Lagerstätte (Yu’anshan Formation, equivalent of the Cambrian Series 2, Stage 3). They typically occur in strongly weathered laminated mudstones. The twenty-four specimens of *S. sinica* studied here are from Jianshan (ELI-0002001, ELI-0002007, ELI-0002008, ELI-0001405, ELI-0002011 to ELI-0002016), Erjie (ELI-0002002, ELI-0002005, ELI-0002009, ELI-0002010, ELI-0002017 to ELI-0002023), Shankou (ELI-0002003), Yunlongsi (ELI-0002004), and Ercaicun (ELI-0002024). The six specimens of *Selkirkia transita* sp. nov. were collected from Erjie (ELI-0000601), Ercaicun (ELI-0000602) and Jianshan (ELI-0000603 to ELI-0000606). Details on the location and lithology of these localities are given in Luo et al. [18]. All specimens are preserved as compressions. Details of their external (e.g. tube) and internal anatomy (e.g. ornamented introvert, gut, oocytes) are typically highlighted by reddish and brownish iron oxides that sharply contrast with the surrounding beige or yellowish matrix.

### Measurements

The tube of *Selkirkia sinica* was measured (length of complete tube) under a Leica M205C binocular microscope using LAS V4.5 software) in 12 specimens from Yunlongsi, 18 from Jianshan, and 90 from Erjie, that of *Selkirkia transita* sp. nov. in 2 specimens from Erjie, 1 from Ercaicun, and 2 from Jianshan (see Additional file 2: Excel S1).

### Photography, scanning electron microscopy and energy-dispersive X-ray spectroscopy

Light photographs were taken with a Canon EOS 5DS R (Northwest University, Xi’an) and Canon EOS 5D Mark IV (University of Lyon 1, digital camera). Micro X-ray Fluorescence (μ-XRF; Northwest University, Xi’an). Elemental mapping was performed with a FEI Quanta 250 FEG at CTμ (University Claude Bernard Lyon 1).

### Phylogenetic analyses

Our cladistic investigation is based on discrete morphological data modified from Harvey et al. [71] and Zhang et al. [10], which focused on cycloneuralian relationships (Additional file 4: Text S3; Additional file 3: Excel S2). *S. willoughbyi* and *S. spencei* were excluded due to a very large amount of uncertain coding [16]. While revising this matrix, we realized that a lot of multistate characters lacked a corresponding neomorphic or “sovereign” binary character (the “sovereign” character being a neomorphic character with explicit dependencies, *sensu* ref. [36]). The inclusion of an “absence” state in a multistate character is justified only if there are reasons to think that the presence of the character did not likely have a single origin; otherwise, the lack of a sovereign character represents an obvious loss of phylogenetic signal. We therefore scrutinized the optimization of relevant character states with Mesquite v.3.61 [72], but found that, in most cases, there was no strong homoplastic pattern justifying the omission of sovereign binary characters. The inclusion of such characters added an important phylogenetic signal to the matrix, but also resulted in less-resolved topologies in parsimony. Because some of this information is conflictual or uncertain in fossils—however, the sovereign characters must necessarily be included.

Parsimony analyses were performed with TNT v.1.5 [73] as well as the R* [74] package TreeSearch v.0.4.3 [75], which uses MorphyLib [76] to handle inapplicable data [77], and Bayesian analyses with MrBayes v.3.2.6 [78]. *Lepidodermella squamata* (Gastrotricha) was chosen as the outgroup, and characters were unordered. In general, higher nodes are very poorly supported and reaching convergence in any method (except heuristic tree bisection reconnection (TBR)) required a greater-than-usual number of replications/generations for a dataset that size.

For parsimony, heuristic TBR was performed with 10 trees saved for 1000 replications. Tree search with new technology (TNT) used default parameters with sectorial search, 1000 iterations of parsimony ratchet, 100 cycles of drifting, and 5 rounds of tree fusing. Owing to the lack of proper handling of inapplicable states by TNT or other common parsimony software (heuristic TBR and TNT treating here inapplicable entries as uncertainties), which could arguably be a significant issue in our matrix (inapplicability representing 20.4% of the data), we decided to also use the TreeSearch package in R*, which makes use of the “scanning” algorithm proposed by [77]. Because an informed use of implied weighting was advocated to yield optimal trees when using parsimony as a conceptual basis [38], we also computed a consensus of separate implied-weighting analysis from TreeSearch using concavity constants 3, 5 and 10, following the recommendations of Smith (2019) [38]. In each case, searches went through 2000 iterations of ratchet.

Bayesian searches used an Mkv+Γ model [79] with 4 runs each with 4 chains for 10,000,000 (*n*=5) generations and burn-in at 20%, which was enough to reach convergence in each case. In light of parsimony results, a backbone was also used to enforce a model with basal Loricifera + Kinorhyncha, using the same procedure.

## Supplementary Information

**Additional file 1: Text S1.** Palaeogeographic distribution of *Selkirkia*. **Figure S1.** Large concentration of *Selkirkia sinica* on a bedding plane, ELI-0002025. White arrow indicates possible bradoriid or brachiopod shell. Scale bar represents 5 mm. **Table S1.** Comparisons between *S. sinica*, *S. transita* sp. nov. and *S. columbia* using nine key characters. **Text S2.** Terminology. **Figure S2.** Pharyngeal teeth types in *Selkirkia* and extant priapulid. **a-c**, **f** *Selkirkia sinica*, general view of ELI-0002001, close-up of everted pharynx, SEM image of pharyngeal tooth (from **b**), and outline. **d**, **e**, **g** *Selkirkia transita* sp. nov., general view of ELI-000601, close-up of everted pharyngeal teeth, outline of pharyngeal tooth (from **e**). **h-j** *Selkirkia columbia*, general view of USNM 83941A (courtesy Jean-Bernard Caron), outline of two types of pharyngeal teeth, modified from Smith et al. [21]. **k**-**m** *Halicryptus spinulosus*, general view (white arrow indicates the everted pharynx) and SEM image showing everted pharynx and tooth. Scale bars represent: **h** 1 cm; **a**, **d**, **k** 5 mm; **l** 1 mm; **b**, **e** 200 μm; **m** 50 μm; **c** 2 μm. **Figure S3.** Weak annulations on the cuticle of *Selkirkia sinica*. **a-c** ELI-0002026, showing general view, close-up and line drawing. **d-f** ELI-0002027, showing general view, close-up and line drawing. Abbreviations: an, annulation; sc, scalid; tc, trunk cuticle; tu, tube. Scale bars represent: **a**, **d** 2 mm; **e** 1 mm; **b** 0.3 mm. **Figure S4.** Consensus cladograms of parsimony. **a, b** Frequency differences consensus topologies. **a** Heuristic Tree Bisection Reconnection only, 34 Most Parsimonious Trees (MPTs), 253 steps. **b** Tree search using new technology (TNT), including Ratchet and Drifting, 7 MPTs, 253 steps. **c** Majority rule consensus. Unweighted analysis using TreeSearch, 277 MPTs, 267 steps. **d** Consensus of TreeSearch analyses using implied weighting for k=(3, 5, 10). Bold branches are extant. Numbers at nodes are jackknifed support values. **Figure S5.** Maximum clade compatibility tree from a Bayesian analysis using an Mkv+Γ model and not a backbone used. **a** Full tree. *Selkirkia* is here resolved as the closest clade to crown-group Scalidophora. Bold branches are extant lineages. Numbers next to node are posterior probabilities. **b** Simplified topology showing the position of *Selkirkia* and harmonic mean (-1,083.58). Dashed lines mark branches supported by extremely low posterior probabilities (≤0.1). **Figure S6.** Brachiopod epibionts on the trunk of *Cricocosmia* (Scalidophora) from the Chengjiang Lagerstätte. ELI-0001200, Jianshan, Haikou. Scale bar represents: 2 mm. **Figure S7.** Polar-coordinate diagram to show the quincunxes distribution of scalids (green dots) as in *Selkirkia* and fossils within crown priapulids (e.g. *Paratubiluchus*).

**Additional file 2: Excel S1.** Measurement of the complete tube of *Selkirkia sinica* and *Selkirkia transita* sp. nov.

**Additional file 3: Excel S2.** Matrix of dataset and Bayesian_harmonic mean.

**Additional file 4: Text S3.** Character description.

## Data Availability

All data generated or analysed during this study are included in this published article [and its supplementary information files].
