# Author Correction to: Tube-dwelling in early animals exemplified by Cambrian scalidophoran worms

**DOI:** 10.1186/s12915-022-01236-z

**Published:** 2022-02-01

**Authors:** Deng Wang, Jean Vannier, Cédric Aria, Jie Sun, Jian Han

**Affiliations:** 1 Shaanxi Key Laboratory of Early Life and Environments, State Key Laboratory of Continental Dynamics, Department of Geology, Northwest University, Xi’an, 710069 China; 2 Univ Lyon, Univ Lyon 1, ENSL, CNRS, LGL-TPE, F-69622 Villeurbanne, France; 3 Shaanxi Key Laboratory of Early Life and Environments, State Key Laboratory of Continental Dynamics, Department of Geology, Northwest University, Xi’an, 710069 China

**Correction to: BMC Biol 19, 243 (2021)**

**https://doi.org/10.1186/s12915-021-01172-4**

The original article [1] erroneously mentioned that *Sullulika* species was from Sirius Passet Lagerstätte.

The authors wish to clarify that it is instead from the Buen Formation in North Greenland.

## References

[1] Wang D (2021). Tube-dwelling in early animals exemplified by Cambrian scalidophoran worms. BMC Biol.

