# Supplementary material for: Tube-dwelling in early animals exemplified by Cambrian scalidophoran worms
Source: BMC Biol. 2021 Nov 12;19:243. doi: 10.1186/s12915-021-01172-4 (PMC8588615; doi:10.1186/s12915-021-01172-4)
Supplement: Supplementary file 1 — Additional file 1: Text S1. Palaeogeographic distribution of Selkirkia. Figure S1. Large concentration of Selkirkia sinica on a bedding plane, ELI-0002025. White arrow indicates possible bradoriid or brachiopod shell. Scale bar represents 5 mm. Table S1. Comparisons between S. sinica, S. transita sp. nov. and S. columbia using nine key characters. Text S2. Terminology. Figure S2. Pharyngeal teeth types in Selkirkia and extant priapulid. a-c, f Selkirkia sinica, general view of ELI-0002001, close-up of everted pharynx, SEM image of pharyngeal tooth (from b), and outline. d, e, g Selkirkia transita sp. nov., general view of ELI-000601, close-up of everted pharyngeal teeth, outline of pharyngeal tooth (from e). h-j Selkirkia columbia, general view of USNM 83941A (courtesy Jean-Bernard Caron), outline of two types of pharyngeal teeth, modified from Smith et al. [21]. k-m Halicryptus spinulosus, general view (white arrow indicates the everted pharynx) and SEM image showing everted pharynx and tooth. Scale bars represent: h 1 cm; a, d, k 5 mm; l 1 mm; b, e 200 μm; m 50 μm; c 2 μm. Figure S3. Weak annulations on the cuticle of Selkirkia sinica. a-c ELI-0002026, showing general view, close-up and line drawing. d-f ELI-0002027, showing general view, close-up and line drawing. Abbreviations: an, annulation; sc, scalid; tc, trunk cuticle; tu, tube. Scale bars represent: a, d 2 mm; e 1 mm; b 0.3 mm. Figure S4. Consensus cladograms of parsimony. a, b Frequency differences consensus topologies. a Heuristic Tree Bisection Reconnection only, 34 Most Parsimonious Trees (MPTs), 253 steps. b Tree search using new technology (TNT), including Ratchet and Drifting, 7 MPTs, 253 steps. c Majority rule consensus. Unweighted analysis using TreeSearch, 277 MPTs, 267 steps. d Consensus of TreeSearch analyses using implied weighting for k=(3, 5, 10). Bold branches are extant. Numbers at nodes are jackknifed support values. Figure S5. Maximum clade compatibility tree from a Bayesian analysis usi [file 12915_2021_1172_MOESM1_ESM.docx]

**Additional files**

**Text S1: Palaeogeographical distribution of *Selkirkia***

*Selkirkia* is represented by the following five species: *Selkirkia* *columbia* from British Columbia, Canada [4], *Selkirkia spencei* and *Selkirkia willoughbyi* from Utah [16], *Selkirkia sinica* [17, 18] and *Selkirkia transita* sp. nov. both from south China, and three undetermined species (*Selkirkia* sp.) from Spence Shale, Utah [16] and the Burgess Shale, British Columbia, Canada [14, 21]. Current paleogeographic reconstructions for the Cambrian ([22], text-figures 2.7, 2.8, 2.9) place the South China plate separated from Laurentia by thousands of kilometres during the early to middle Cambrian interval (over 10 Ma). The presence of *Selkirkia* on two distant plates would imply faunal migration or dispersal. Considering that *Selkirkia* had limited capacities for moving over long distances, egg or larval dispersion by currents seems to best explain their palaeogeographic distribution. In modern endobenthic priapulids fertilization is external and followed by the development of hatching and loricate larvae that can be easily transported by currents. For example, *Priapulus caudatus* is found on either side of the Atlantic Ocean (Sweden, [23]; USA, [24]), the Pacific Ocean (Alaska; [25], the White Sea in Russia [26] and Antarctic (Chile, van der [11]).

**
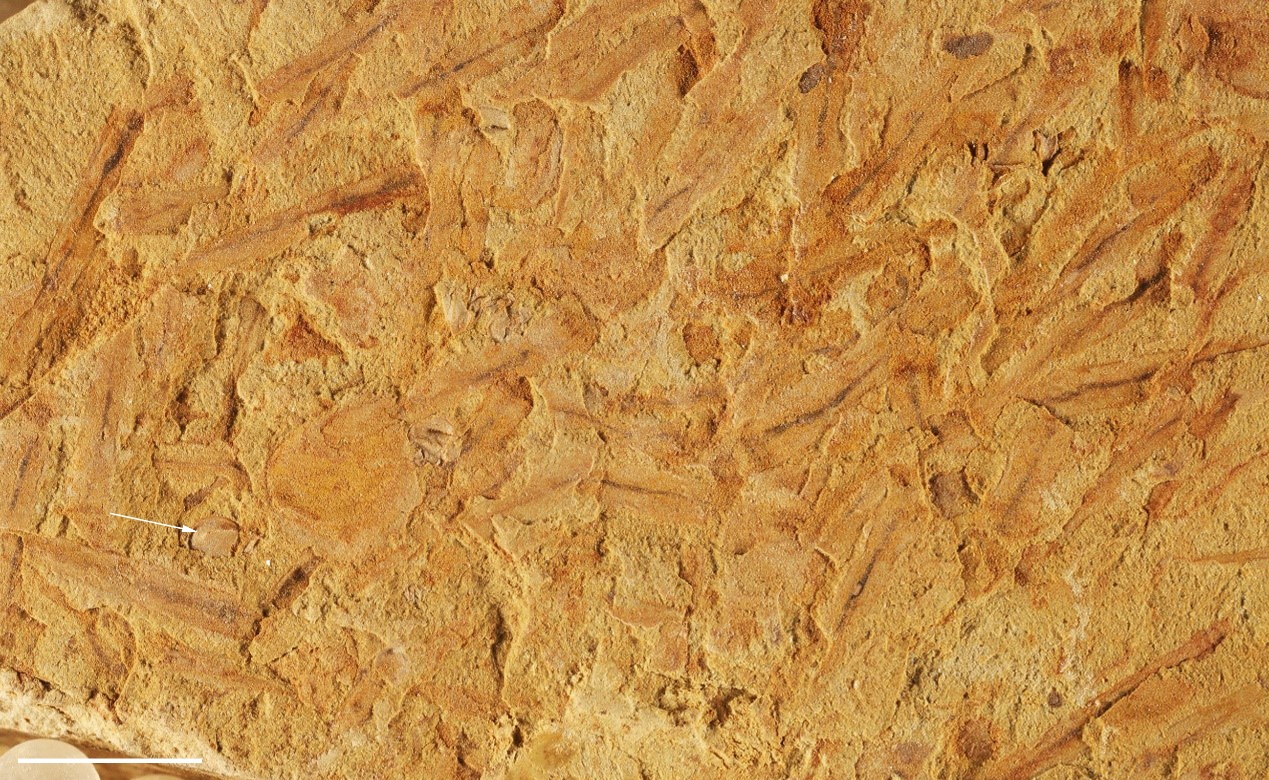
**

**Figure S1**. **Large concentration of *Selkirkia sinica* on a bedding plane, ELI-0002025**. Arrow indicates possible bradoriid or brachiopod shell. Scale bar represents 5 mm.


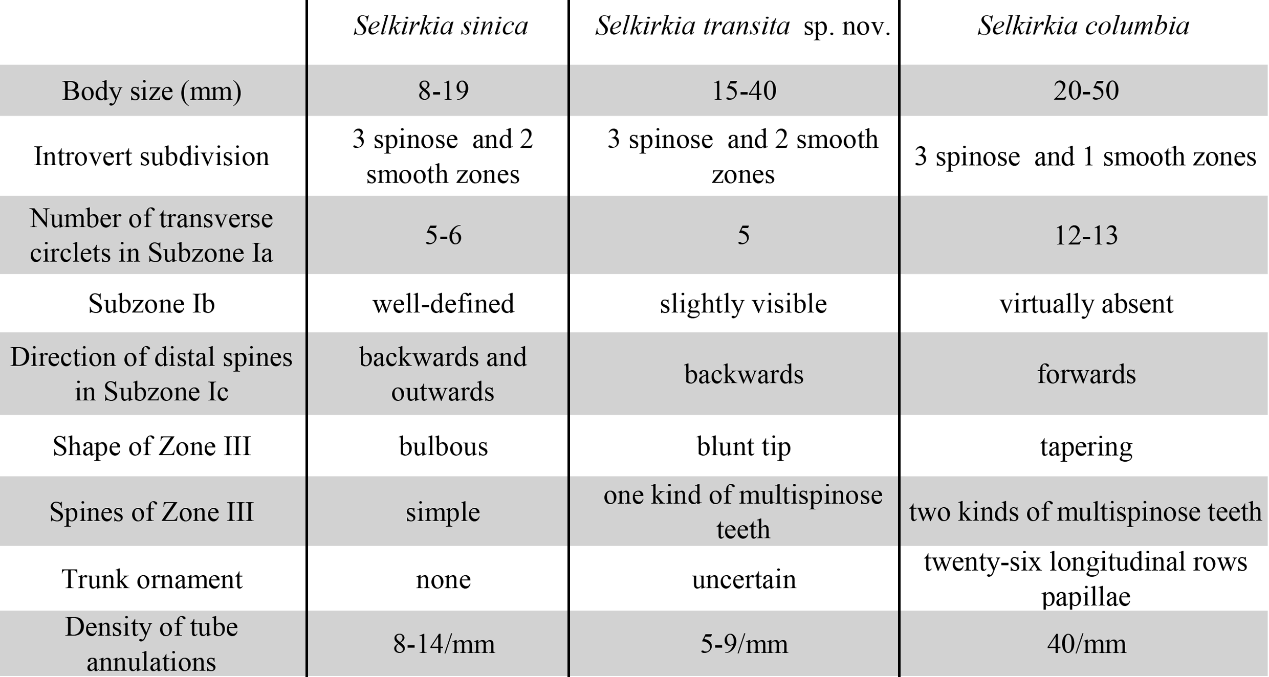


**Table S1**. **Comparisons between *S. sinica*, *S. transita* sp. nov. and *S. columbia* using nine key characters.**

**Text S2: Terminology**

Proboscis and introvert are very often used interchangeably in the scientific literature to designate the everted part of scalidophoran worms. According to Schmidt-Rhaesa [2], the body of priapulids, kinorhynchs and loriciferans is divided into a trunk region and an introvert defined as the body part which “*can be withdrawn completely into the trunk by the action of retractor muscles*” [2], p.147). The pressure exerted by trunk muscles on the fluid contained in the blastocoelomic cavity is responsible for the eversion of the introvert. In its fully everted state, the introvert generally exhibits, from proximal to terminal, a swollen and spiny region covered by scalids (spine-like cuticular structure) followed by the pharynx lined with teeth. In contrast, the term “proboscis” lacks a precise definition and is unspecific among invertebrates (Schmidt-Rhaesa, personal communication). For these reasons, we prefer to use “introvert” instead of “proboscis” in our morphological descriptions. We followed the terminology (Zones I to III) used by Conway Morris [4] to describe the introvert of *Selkirkia* and added additional subdivisions.


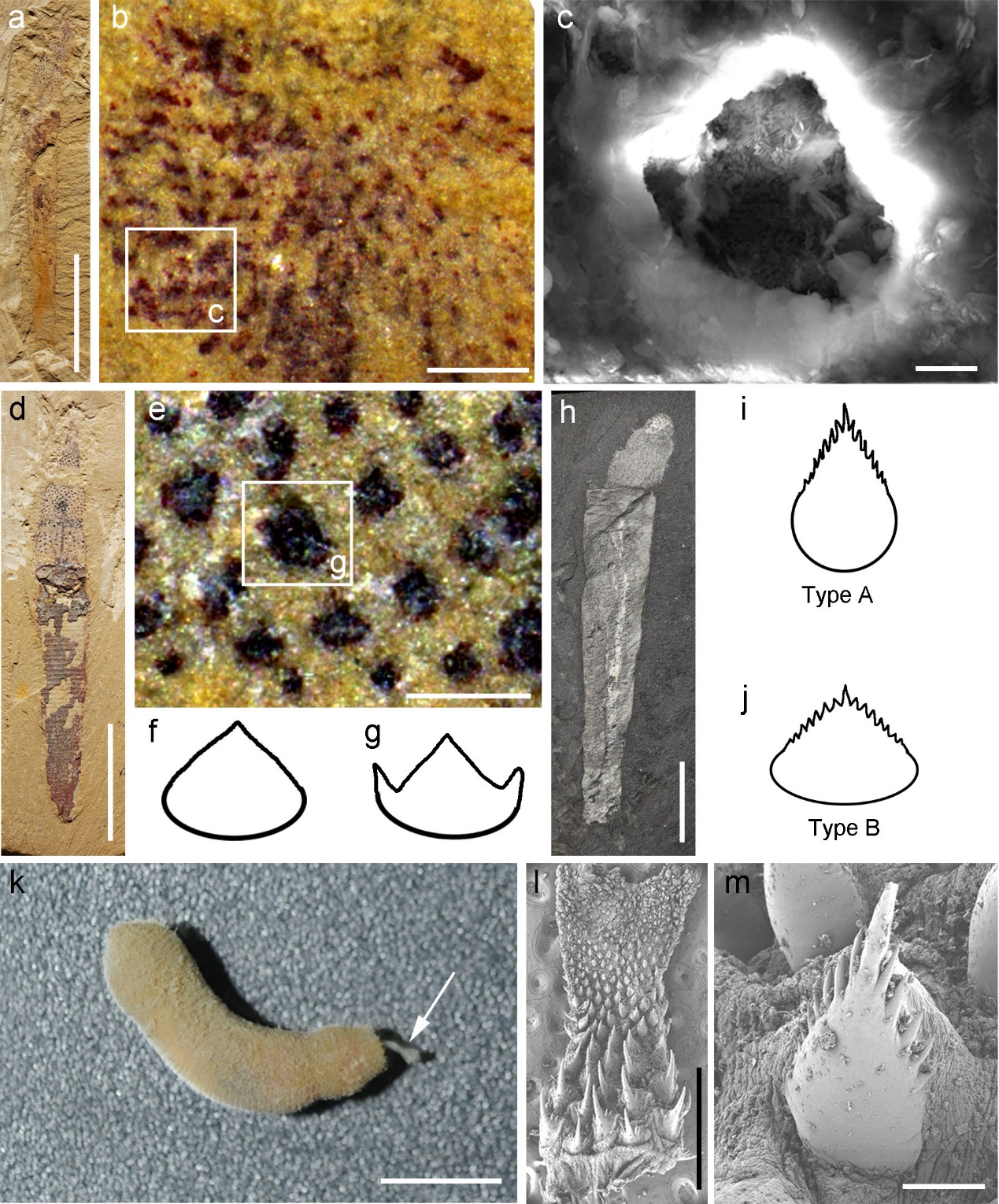


**Figure S2. Pharyngeal teeth types in *Selkirkia* and extant priapulids**. **a**-**c**, **f** *Selkirkia sinica*, general view of ELI-0002001, close-up of everted pharynx, SEM image of pharyngeal tooth (see location in **b**), and outline. **d**, **e**, **g** *Selkirkia transita* sp. nov., general view of ELI-000601, close-up of everted pharyngeal teeth, outline of pharyngeal tooth (see location in **e**). **h**-**j**, *Selkirkia columbia*, general view of USNM 83941A (courtesy of Jean-Bernard Caron), outline of two types of pharyngeal teeth, modified from Smith et al. (2015). **k**-**m**, *Halicryptus spinulosus*, general view (white arrow indicates the everted pharynx) and SEM image showing everted pharynx and tooth. Scale bars represent: **h**, 1 cm; **a**, **d**, **k**, 5 mm; **l**, 1 mm; **b**, **e**, 200 μm; **m**, 50 μm; **c**, 2 μm.


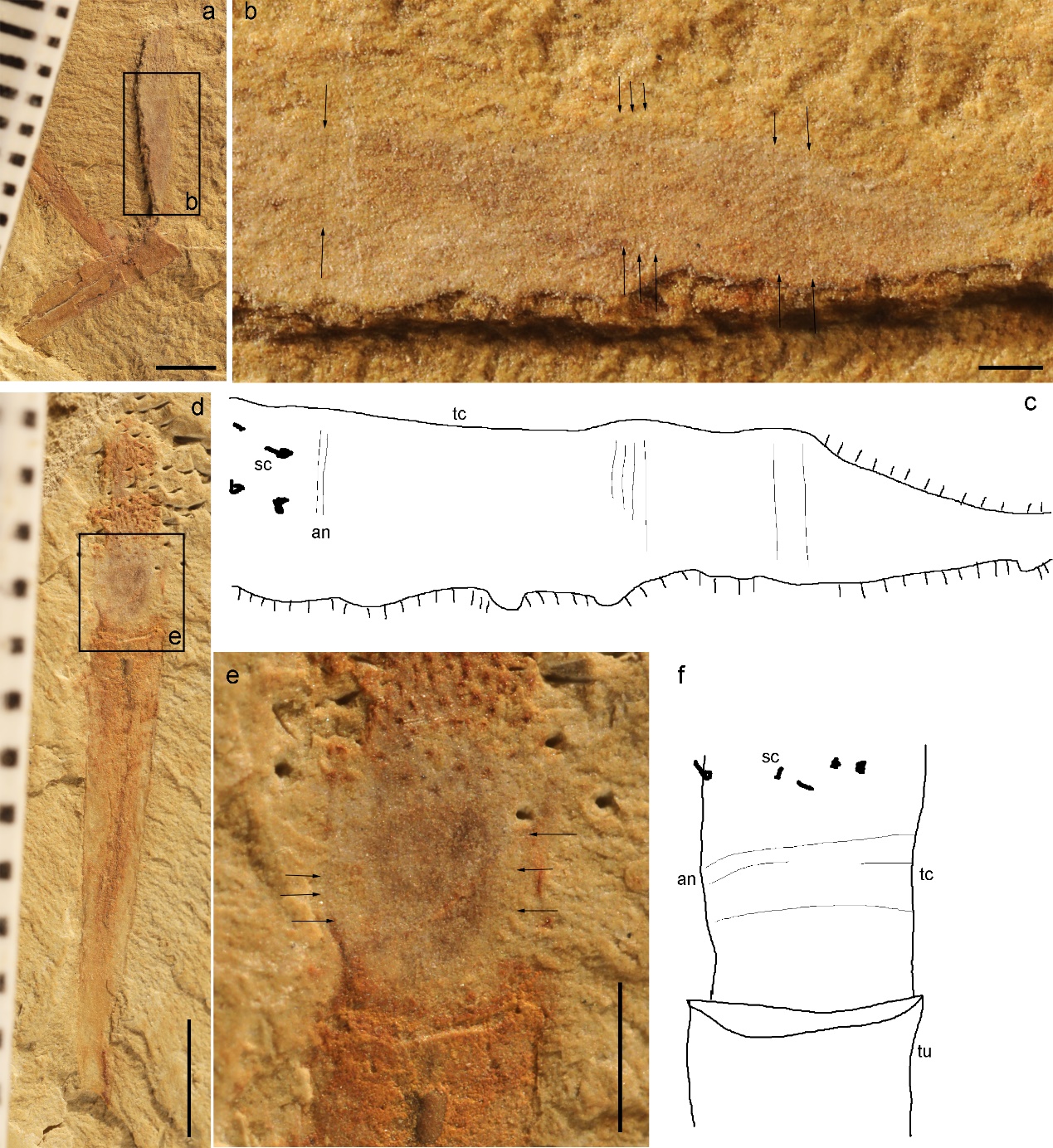


**Figure S3**. **Weak annulations on the cuticle of *Selkirkia sinica***. **a-c** ELI-0002026, general view, close-up and line drawing. **d-f** ELI-0002027, general view, close-up and line drawing. Abbreviations: an, annulation；sc, scalid；tc, trunk cuticle；tu, tube. Scale bars represent: **a**, **d**, 2 mm; **e**, 1 mm; **b**, 0.3 mm.


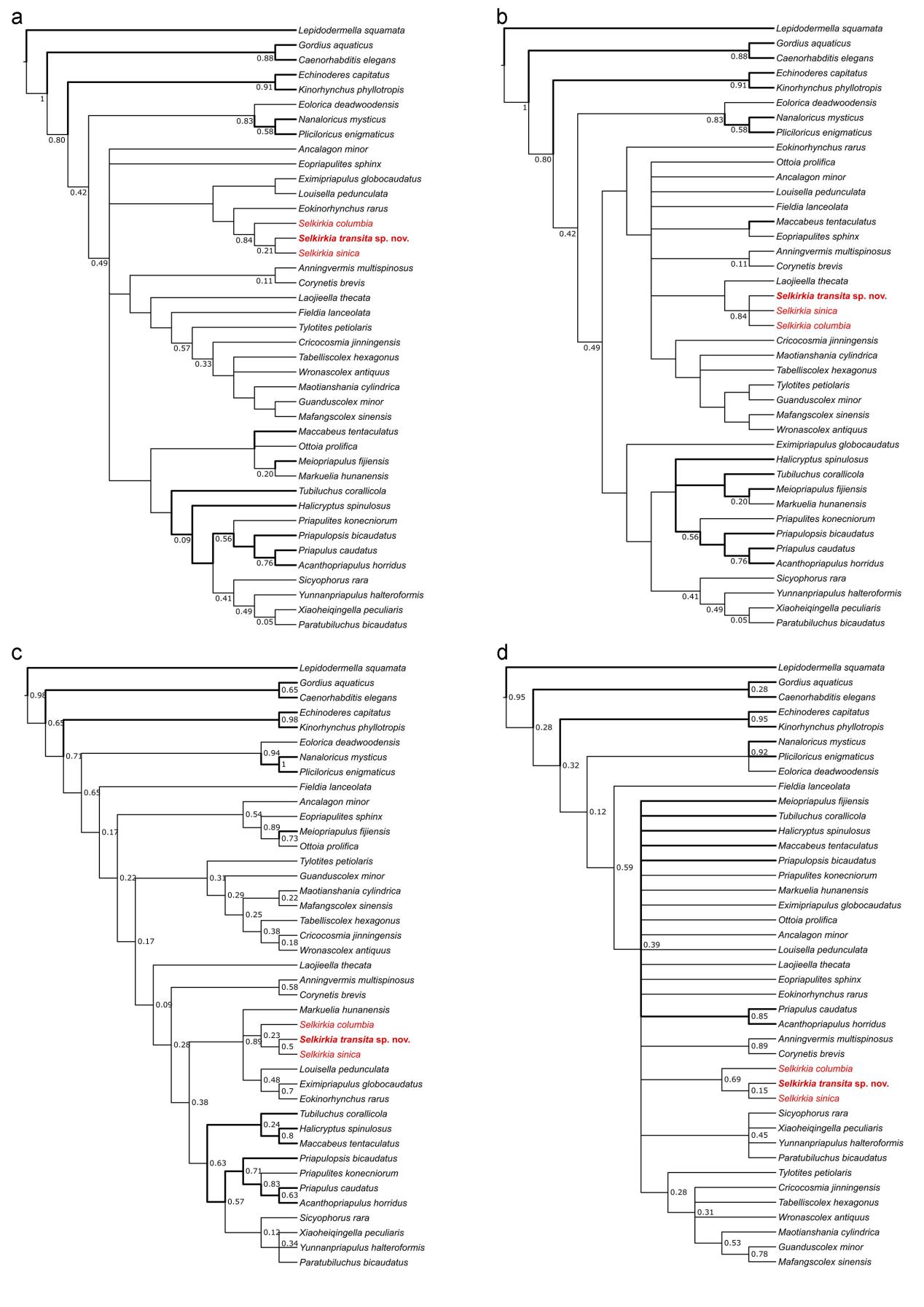


**Figure S4**. Consensus cladograms of parsimony. **a** Heuristic Tree Bisection Reconnection, 34 Most Parsimonious Trees (MPTs), 253 steps. **b** Tree search using new technology (TNT), including Ratchet and Drifting, 7 MPTs, 253 steps. **c** Unweighted analysis using TreeSearch, 277 MPTs, 267 steps. **d** Consensus of TreeSearch analyses using implied weighting for k=(3, 5, 10). Bold branches are extant. Numbers at nodes are jackknifed support values; italicized numbers at nodes in A and B are frequencies of occurrence for nodes not present in all MPTs.


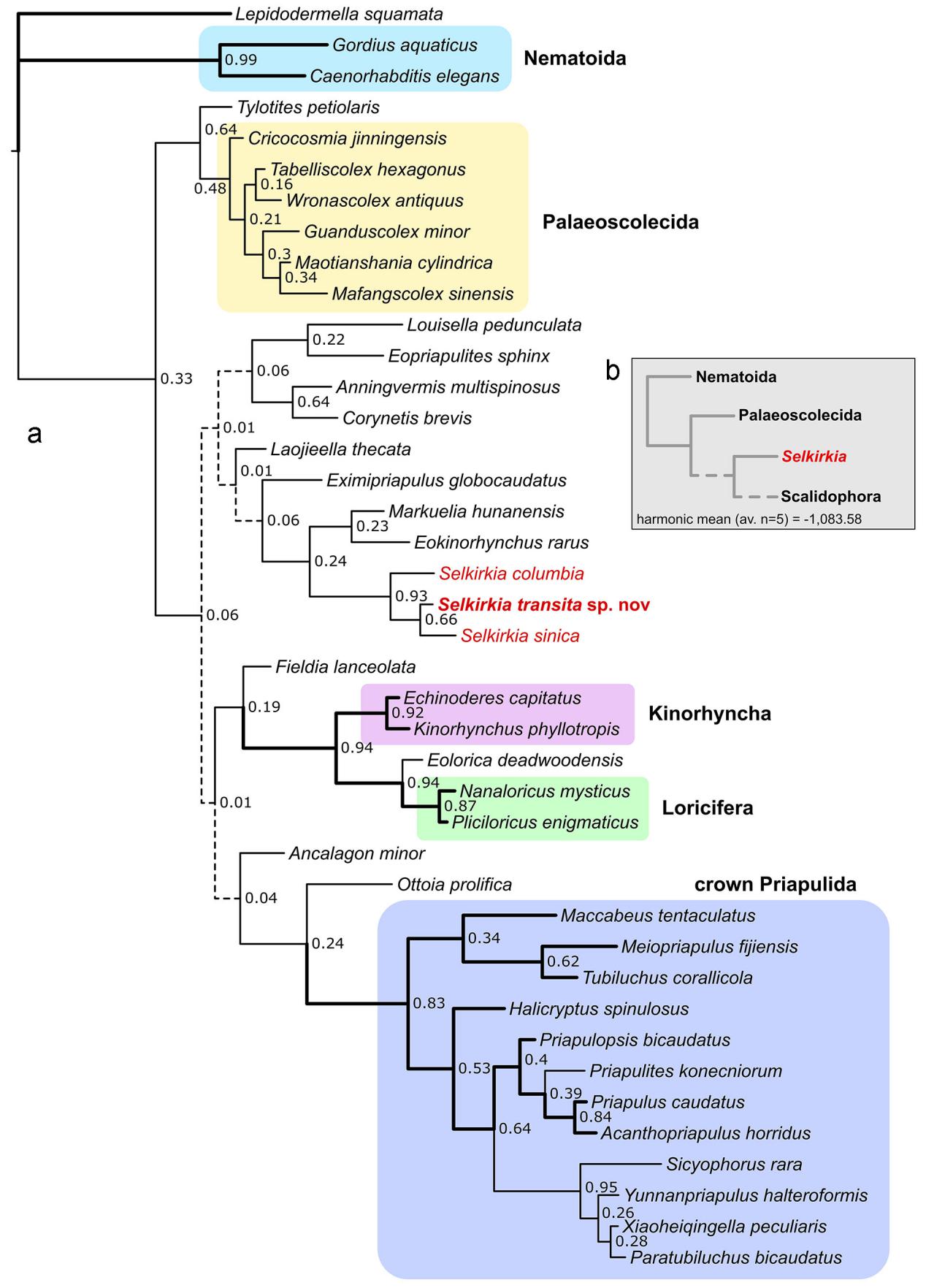


**Figure S5**. Maximum clade compatibility tree from a Bayesian analysis using an Mkv+Γ model and not a backbone used. **a** Full tree. *Selkirkia* is here resolved in the basalmost clade within total-group Scalidophora. Bold branches are extant species. Numbers next to node are posterior probabilities. **b** Simplified topology showing the position of *Selkirkia* and harmonic mean (-1,083.58). Dashed lines indicate alternative placements of *Eximipriapulus* at 10 million generations.


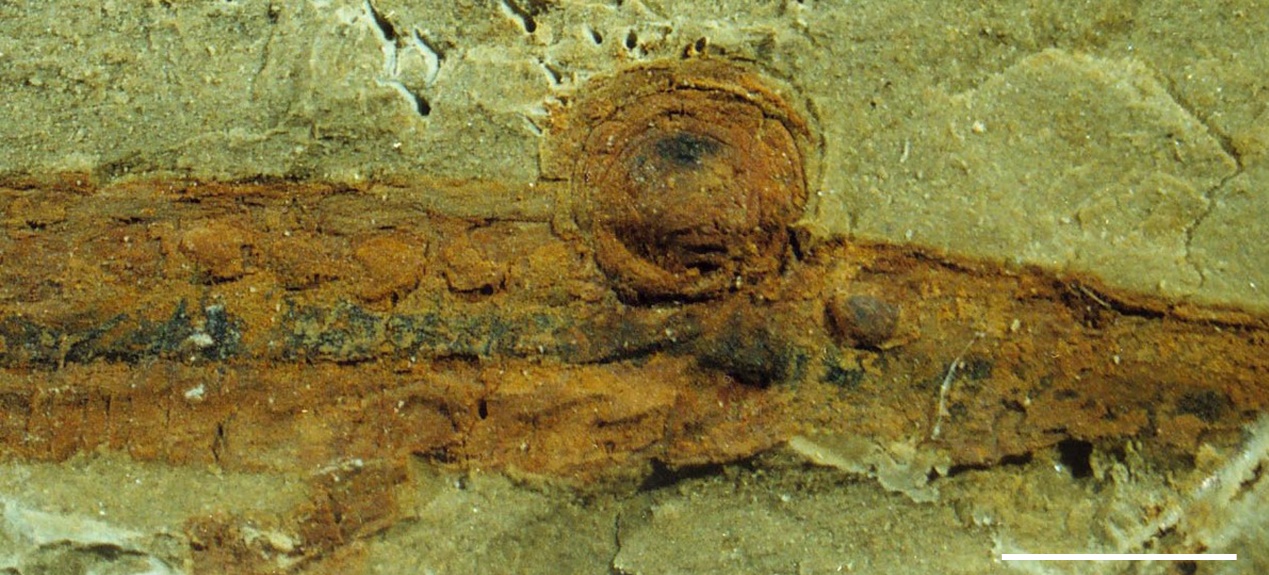


**Figure S6.** T**runk of *Cricocosmia* from the Chengjiang Lagerstätte, showing an attached brachiopod shell to the worm’s cuticle**. ELI-0001200, Jianshan, Haikou. Scale bar represents: 2 mm.


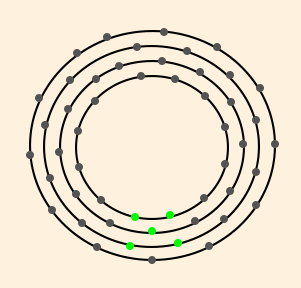


**Figure S7**. Polar-coordinate diagram showing the distribution in quincunx of 25 longitudinal rows of scalids (green dots) as in *Selkirkia* and fossils within crown priapulids (e.g. *Paratubiluchus*).
