# Supplementary material for: Tube-dwelling in early animals exemplified by Cambrian scalidophoran worms
Source: BMC Biol. 2021 Nov 12;19:243. doi: 10.1186/s12915-021-01172-4 (PMC8588615; doi:10.1186/s12915-021-01172-4)
Supplement: Supplementary file 4 — Additional file 4: Text S3. Character description. [file 12915_2021_1172_MOESM4_ESM.docx]

**Characters description**

**General body plan in adults**

1. Body shape:

(0) cylindrical,

(1) gourd-like,

2. Type of anterior termination of body:

(0) ‘head’,

(1) introvert.

Note: Status (0), a head appears of Nematoida and Gastrotrich which cannot everted and overall is no unambiguous boundary with trunk but bearing a few cuticular elements (Brusca et al., 2016). Head of Nematoida can be considered as Zone I which is defined in Scalidophora.

3. Terminal mouth opening:

(0) visible or closed,

(1) with stiff cilia,

(2) with amphids,

(3) with oral elements of mouth cone,

(4) with teeth of pharynx.

4. Percentage of animal length taken up by introvert:

(0) between 1% and 30%,

(1) 31 to 50%.

5. Body cavity:

(0) absent

(1) present.

6. Body cavity: type

(0) largely filled by mesenchyme and organs,

(1) forms a blastocoel cavity

7. Body musculature:

(0) only longitudinal muscles,

(1) longitudinal and circular muscles.

**Head /introvert**

8. Head: cuticular ornaments

(0) absent,

(1) present.

9. Degree to which the Zone I can be invaginated:

(0) not invaginable at all,

(1) partially invaginable,

(2) completely invaginable into the trunk.

10. Zone I: overall shape

(0) cylindrical,

(1) swollen.

11. Zone I: number of subdivisions

(0) 1,

(1) 2,

(2) 3.

Note: The introvert of scalidophoran worms can be divided into three parts named (from

proximal to distal): Zone I (with scalids), Zone II (between Zone I and Zone II), and Zone III corresponding to the everted pharynx) (Conway Morris, 1977).

12. Subzone Ia: cuticular elements

(0) absent

(1) present

Note: “Subzone Ia” defined in main text represents the proximal part of Zone I (e.g. *Selkirkia*, *Louisella*, *Eokinorhynchus*). When the species have no subdivisions of Zone I (i.e Char. 12 status (0), e.g. *Priapulus*, *Maotianshania*), then “Subzone Ia” is equal to Zone I, thus the codings of Subzones Ib, Ic of these species are coded as inapplicable (-) (i.e. Chars 23~25).

13. Subzone Ia: regions covered by cuticular elements

(0) whole region

(1) only anterior part

Note: Cuticular ornaments of some species such as *Sicyophorus* and *Cricocosmia* only cover the anterior part of Subzone Ia.

14. Subzone Ia: scalids distributed along longitudinal ridges

(0) absent

(1) present

15. Subzone Ia: distribution pattern of cuticular elements

(0) no visible regular pattern,

(1) discrete parallel longitudinal rows,

(2) quincunx.

16. Subzone Ia: radial distribution of cuticular elements

(0) pentaradial,

(1) hexaradial.

17. Subzone Ia: morphology of cuticular elements

(0) papillae,

(1) simple spines or conical scalids,

(2) hooks or spinose hooks,

(3) telescopiform scalids,

(4) curved scalids and dentoscalids,

(5) complex scalids,

(6) glandular scalids, trifid spines, sensory spines,

(7) scalids with pectinate hood,

(8) spinoscalids and trichoscalids,

(9) spinoscalids and clavoscalids.

18. Subzone Ia: number of trichoscalids

(0) 6,

(1) 14.

19. Subzone Ia: number of longitudinal rows of elements

(0) 6-17,

(1) 20,

(2) 18,

(3) 25,

(4) 28,

(5) 30,

(6) > 31.

20. Subzone Ia: sequence of scalids

(0) elements organized in a single series,

(1) elements organized in two or more series.

21. Subzone Ia: basal circlet

(0) no constriction or longitudinal or circular muscles,

(1) marked by a constriction, longitudinal or circular muscles.

22. Subzone Ib:

(0) absent

(1) present

Note: “Subzone Ib” defined in main text represents the middle smooth part of Zone I.

23. Subzone Ib: length

(0) almost invisible

(1) narrow

(2) wide

24. Subzone Ic:

(0) absent

(1) present with simple spinose scalids

Note: “Subzone Ic” defined in main text represents the distal part of Zone I.

25. Zone II: cuticular ornament

(0) none,

(1) few cuticular elements (e.g. tiny spines, papillae).

26. Anteriormost of Zone I or basal Zone II in adults: anteriorly pointing of elongate basal spines/coronal scalids

(0) absent,

(1) present.

27. Zone III type:

(0) retractable mouth cone bearing oral elements,

(1) eversible pharynx bearing teeth.

Note: “Zone III” defined in main text represents pharynx located at the distal part of introvert. Mouth cone is present in Recent Kinorhyncha and Loricifera within Scalidophora. It may correspond to the division of Zone III of Priapulida.

**Pharyngeal teeth/oral elements**

28. Zone III: cuticular elements

(0) spines,

(1) multispinose,

(2) hooks,

(3) conical with a fringe of spines,

(4) sclerotized trabeculae,

(5) pectinate.

Note: Zone III in Characters 29~32 includes eversible pharynx bearing teeth.

29. Zone III: number of elements in basal circlet

(0) 5,

(1) 18,

(2) 10,

(3) 15,

(4) 25,

(5) 16,

(6) 30.

30. Zone III: eversibility

(0) completely eversible,

(1) incompletely eversible, but eversible beyond the proximal teeth,

(2) normally eversible only as far as the proximal teeth.

31. Zone III: size evenness of cuticular elements from proximal to distal

(0) approximately equal size,

(1) decrease,

(2) increase.

32. Zone III: type of oral elements

(0) cuticular ridges,

(1) styles.

Note: Zone III in characters 33~34 represents retractable but inversible mouth cone bearing oral elements (refer to Char. 8) differing from the manner as the pharynx of Priapulida. Here mouth cone is only for Kinorhyncha and Loricifera.

33. Zone III: number of outer oral elements

(0) 6,

(1) 8,

(2) 9.

34. Pharyngeal lumina:

(0) round,

(1) triradiate

(2) triradiate in the distal and hexagonal in the proximal region.

Note: Pharyngeal lumina is present in Cycloneuralia and Gastrotricha.

35. Two rings of pharynx retractors penetrating the brain:

(0) absent

(1) present

Note: This is an autapomorphy of Scalidophora (Nielsen 2012).

36. Internal structure of cuticular elements of anterior termination of body:

(0) composed exclusively of cuticle (e.g. amphids),

(1) cuticle limited to a thin outer covering (e.g. scalids and sensory cilia).

37. Rings of cuticular elements of anterior termination of body:

(0) absent

(1) present

Note: An autapomorphy of Scalidophora is present as the rings of pharyngeal teeth (Priapulida) or oral elements of mouth cone (Kinorhyncha and Loricifera) (Nielsen 2012).

**Neck in adults- (trunk-introvert boundary)**

38. Neck:

(0) absent,

(1) present.

39. Neck cuticular ornament:

(0) cuticular placids,

(1) cuticular plates + scalids,

(2) transverse wrinkles with cuticular spines,

(3) cuticular scalids or papillae.

40. Number of placids:

(0) 8,

(1) 16.

**Trunk in adults**

41. Trunk length to width ratio:

(0) <10,

(1) between 10 and 20,

(2) >20.

42. Annulations:

(0) absent,

(1) present.

43. Number of trunk annulations:

(0) 12-29,

(1) 30-60,

(2) 61-139,

(3) 140 or more.

44. Trunk segmentation:

(0) absent,

(1) present.

45. Trunk segment 1:

(0) ring-like,

(1) with 3 sternal + 1 tergal plates.

46. Cuticular elements:

(0) absent,

(1) present.

47. Abundance of cuticular elements

(0) no cuticular element abundantly covering the body

(1) at least one type largely covering the body

48. Most cuticular elements covering whole trunk: morphology

(0) sclerites,

(1) spines,

(2) round or oval plates,

(3) longitudinal arranged plates/plicae,

(4) areoles,

(5) cilia,

(6) tumuli,

(7) tubuli,

(8) tubercles.

49. Trunk papillae:

(0) absent,

(1) present.

50. Trunk papillae: morphology

(0) simple,

(1) extremely long.

51. Trunk papillae: location

(0) in ring at posterior trunk,

(1) scattered distribution,

(2) in ring at anterior trunk,

(3) in two longitudinal rows at middle part of ventral trunk.

52. Small nodes/tubercles on surface of plates or sclerites:

(0) absent,

(1) present.

Note: Nodes/tubercles are present only on the surface of palaeoscolecidan plates or sclerites of *Tabelliscolex*, respectively.

53. Number of transverse rows of cuticular elements present in each annulation:

(0) 1,

(1) 2,

(2) 3,

(3) 4,

(4) 5,

(5) irregular pattern,

(6) 3 and 5.

54. Lorica: Number of plates/plicae

(0) 6,

(1) 22,

(2) 20,

(3) 13~15.

55. Trunk tubuli:

(0) absent,

(1) present.

56. Trunk tubercles:

(0) absent,

(1) present.

57. Trunk flosculi, N-flosculi or sensory spots:

(0) absent,

(1) flosculi present (between 7 and 16 petals).

**Around anal region and posterior termination in adults**

58. Spines or hooks around anus in an arc or ring:

(0) absent,

(1) present.

59. Paired tubulae or setae or hooks:

(0) absent,

(1) present.

60. Terminal warts:

(0) absent,

(1) present.

61. Terminal warts: size

(0) 1% to 5% of trunk diameter,

(1) 6% to 10% of trunk diameter.

62. Eversible bursa on the termination of trunk end:

(0) absent

(1) present

63. Caudal appendage(s):

(0) absent,

(1) present.

64. Caudal appendage(s): length

(0) less than the length of the body,

(1) up to three times the length of the body.

65. Caudal appendage(s): number and position

(0) single and positioned terminally,

(1) single and positioned dorso-medially,

(2) bicaudal.

66. Caudal appendage(s): morphology

(0) undivided,

(1) pseudosegmented (external).

67. Caudal appendage: surface

(0) smooth,

(1) vesiculate,

(2) bearing hooks,

(3) bearing transverse wrinkles or striations.

68. Position of the anus:

(0) terminal, whether within bursa or otherwise,

(1) in posterolateral or posteroventral surface of abdomen.

69. Polythyridium:

(0) absent,

(1) present.

**Tube**

70. Tube:

(0) absent,

(1) present.

71. Tube morphology:

(0) composed of agglutinated debris,

(1) cuticular and annulated.

**Nervous system**

72. Unpaired ventral nerve cord throughout its length:

(0) absent,

(1) present.

73. Ventral nerve cords merging caudally:

(0) absent,

(1) present.

74. Circumpharyngeal brain:

(0) absent,

(1) present.

75. Brain with anterio-posterior sequence (perikarya-neuropil-perikarya):

(0) absent,

(1) present.

76. Apical part of brain composed of perikarya:

(0) absent,

(1) present.

77. Pharyngeal nervous system:

(0) absent,

(1) present.

78. Two rings of introvert/head retractors passing through the collar-shaped brain:

(0) absent,

(1) present.

**Larval and Developmental characters**

79. Developmental type:

(0) direct,

(1) biphasic.

80. Loricate larval stage:

(0) absent,

(1) present.

81. Cuticle of the larva/juvenile dorso-ventrally flattened (at least in older stages), with six lateral plates in-folded and accordion like:

(0) absent,

(1) present.

82. Cuticles of the annuli or segments divided into plates, especially in larva/late juveniles:

(0) absent,

(1) present.

83. Six long pharynx retractor muscles in larvae/juveniles:

(0) absent,

(1) present.

84. Differentiated introvert in larva/juvenile:

(0) absent,

(1) present.

**Reproductive system**

85. Cloaca in both sexes:

(0) absent,

(1) present.

86. Protonephridia:

(0) absent,

(1) present.

87. Protonephridia: relation with gonad

(0) do not flow into the gonoduct and are not integrated into the gonad,

(1) flow into the gonoduct and/or are integrated into the gonad.

88. Urogenital system attached to the body wall by a ligament:

(0) absent,

(1) present.

89. Spermatozoa with a flagellum:

(0) absent,

(1) present.

**Cuticle**

90. Cuticle predominantly containing collagen:

(0) absent,

(1) present.

91. Cuticle containing chitin:

(0) absent,

(1) present.

92. Distribution of chitin in cuticle:

(0) predominantly within the middle cuticle layer (exocuticle),

(1) predominantly within the lowermost cuticle layer (endocuticle),

(2) eggshells or pharyngeal cuticle.

93. Cuticle with homogeneous layer at surface/beneath epicuticle:

(0) absent,

(1) present.

94. Exocuticle:

(0) Homogenous,

(1) Heterogenous.

95. Exocuticle: radially striated or vertical canal:

(0) absent,

(1) present.

96. Crisscrossed fibres in cuticle:

(0) absent,

(1) present.
